# Error Estimates and Adaptivity of the Space-Time Discontinuous Galerkin Method for Solving the Richards Equation

**DOI:** 10.1007/s10915-024-02650-x

**Published:** 2024-08-20

**Authors:** Vít Dolejší, Hyun-Geun Shin, Miloslav Vlasák

**Affiliations:** 1 Faculty of Mathematics and Physics, Charles University, Sokolovská 83, Prague, Czech Republic; 2 Faculty of Civil Engineering, Czech Technical University, Thakurova 7, Prague 6, 166 29 Czech Republic

**Keywords:** Space-time discontinuous Galerkin method, Richards equation, A posteriori error estimate, *hp*-mesh adaptation, 65M60, 65M15, 65M50

## Abstract

We present a higher-order space-time adaptive method for the numerical solution of the Richards equation that describes a flow motion through variably saturated media. The discretization is based on the space-time discontinuous Galerkin method, which provides high stability and accuracy and can naturally handle varying meshes. We derive reliable and efficient a posteriori error estimates in the residual-based norm. The estimates use well-balanced spatial and temporal flux reconstructions which are constructed locally over space-time elements or space-time patches. The accuracy of the estimates is verified by numerical experiments. Moreover, we develop the *hp*-adaptive method and demonstrate its efficiency and usefulness on a practically relevant example.

## Introduction

Fluid flows in variably saturated porous media are usually described by the Richards equation [33], which is expressed in the form

1 $$\begin{aligned} \partial _t\vartheta (\psi ) - \nabla \cdot \big ({\textbf{K}}(\theta (\psi )) (\nabla \psi + \nabla z) \big ) = g, \end{aligned}$$

where $\partial _t$ denotes the derivative with respect to time, $\nabla \cdot$ and $\nabla$ are the divergence and gradient operators, respectively, $\psi$ is the sought pressure head (= normalized pressure), *z* is the vertical coordinate, $\theta$ is the water content function, ${\textbf{K}}$ is the hydraulic conductivity tensor and *g* is the source term. In addition, the active pore volume $\vartheta$ is related to $\theta$ by the following relation

2 $$\begin{aligned} {\vartheta }(\psi ) := \theta (\psi ) + \frac{S_s}{\theta _s} \int \nolimits _{-\infty }^{\psi } \theta (s){\,{\mathrm d}s}, \end{aligned}$$

where $S_s,\theta _s\ge 0$ are material parameters. The hydraulic conductivity satisfies ${\textbf{K}}(\psi )= {\textbf{K}}_s {{{\mathcal {K}}}}_r(\psi )$, where ${\textbf{K}}_s$ is the saturated conductivity tensor, and ${{{\mathcal {K}}}}_r\in [0,1]$ is the relative saturation. The functions $\theta$ and ${{{\mathcal {K}}}}_r$ are given by constitutive relations, e.g., by van Genuchten’s law [27] and by Mualem’s law [31], respectively.

The Richards equation belongs to the nonlinear parabolic problems, and it can degenerate, in particular ${\textbf{K}}\rightarrow 0$ or $\tfrac{{\mathrm d}{\vartheta }}{{\mathrm d}\psi }\rightarrow 0$. Due to the degeneracy, the numerical solution is challenging, and various techniques have been developed for its solution in the last decades, see [25] for a survey.

In [14], we presented the adaptive space-time discontinuous Galerkin (STDG) method for the numerical solution of (1). This technique is based on a piecewise polynomial discontinuous approximation with respect to both the spatial and temporal coordinates. The resulting scheme is sufficiently stable, provides high accuracy, and is suitable for the *hp*-mesh adaptation. This is an important property, since the weak solution of the Richards equation is (only) piecewise regular and exhibits singularities along the material interfaces and the unsaturated/saturated zone (when $\psi \approx 0$). Therefore, an adaptive method that allows different meshes at different time levels, can achieve an accurate approximation with a relatively small number of degrees of freedom.

The numerical experiments presented in [14] showed the potential of the adaptive STDG method. However, the mesh adaptation used is based on interpolation error estimates that do not guarantee an upper error bound. The aim of this work is to overcome this bottleneck, derive a posteriori error estimates, and use them in the *hp*-mesh adaptation framework.

A posteriori error estimates for the numerical solution of the Richards equation have been treated in many papers for different numerical methods. We mention the finite volume framework with multistep time discretization in [5], the mixed finite element method in [6], the two-point finite volume discretization in [8], the lowest-order discretization on polytopal meshes in [38], finite element techniques in [30] and the references cited therein.

Guaranteed error estimates without unknown constants are usually obtained by measuring the error in a dual norm of the residual. Introducing reconstructed fluxes from the space $H^1(\textrm{div},{\varOmega })$, the upper bound can then be obtained directly. In [18], we developed this approach to the higher-order STDG method for nonlinear parabolic problems, where the temporal discontinuities were treated by temporal flux reconstructions considering the time jumps.

In this paper, we extend the approach [18] to the Richards equation (1). Although the definition of the temporal and spatial flux reconstructions as well as the derivation of the upper bounds is straightforward, the proof of the lower bound (efficiency) is rather tricky since the term $\theta (\psi )$ in the time derivative is not a polynomial function for a polynomial $\psi$. In contrary to [18], the proof of efficiency requires the additional oscillatory data terms. We construct spatial fluxes by solving local Neumann problems defined on space-time patches that generalize the approach from [22]. Moreover, we provide numerical experiments verifying derived error estimates. Compared to [18], the resulting effectivity indices are much closer to one. This is the first novelty of this paper.

Secondly, we deal with the errors arising due to iterative solution of nonlinear algebraic systems. We introduce a cheap stopping criterion for iterative solvers and justify it by numerical experiments. Thirdly, we introduce a space-time adaptive algorithm that employs the anisotropic *hp*-mesh adaptation technique [15]. The algorithm admits local adaptation of size and shape of mesh elements and the local adaptation of degrees of polynomial approximation with respect to space. However, the size of the time step can vary globally, and the degree of polynomial approximation with respect to time is fixed. Using the equidistribution principle, the algorithm gives an approximate solution with the error estimate under the given tolerance. The performance of the adaptive algorithm is demonstrated numerically, including a practically relevant example.

The rest of the paper is organized as follows. In Sect. 2, we introduce the problem considered, its STDG discretization is briefly described in Sect. 3. The main theoretical results are derived in Sect. 4, where the upper and lower bounds are proved. Two possible spatial reconstructions are discussed in Sect. 5 together with the stopping criteria of iterative solvers. The numerical verification of the error estimates is given in Sect. 6. Furthermore, we present the resulting *hp*-mesh adaptation algorithm in Sect. 7 and demonstrate its performance by numerical examples. Finally, we conclude with some remarks in Sect. 8.

## Problem Formulation

Let ${\varOmega }\subset {\mathbb {R}}^d$ ($d=2,3$) be the domain occupied by a porous medium and $T>0$ the physical time to be reached. For simplicity, we assume that ${\varOmega }$ is polygonal. By ${\varGamma }:=\partial {\varOmega }$, we denote the boundary of ${\varOmega }$ which consists of two disjoint parts: the Dirichlet boundary ${\varGamma _\textrm{D}}$ and the Neumann boundary ${\varGamma _\textrm{N}}$. We write the Richards equation (1) in a different form, which is more suitable for the analysis. We seek a function $u= u(x,t):{\varOmega }\times (0,T)\rightarrow {\mathbb {R}}$, which represents a hydraulic head (with the physical unit $\textrm{L}$). The quantity $u$ is related to the pressure head $\psi$ by $u= \psi + z$. The Richards equation (1) reads

3 $$\begin{aligned}&\partial _t{\vartheta }(u) - \nabla \cdot ({\textbf{K}}(u) \nabla u) = g \quad \text{ in } {\varOmega }\times (0,T) \\&u= u_D \text{ on } {\varGamma _\textrm{D}}\times (0,T) \nonumber \\&{\textbf{K}}(u) \nabla u\cdot n= g_N \text{ on } {\varGamma _\textrm{N}}\times (0,T), \nonumber \\&u(x, 0) = u_0 \ \text{ in } {\varOmega }, \nonumber \end{aligned}$$

where $g:{\varOmega }\times (0,T)\rightarrow {\mathbb {R}}$ represents a source term if *g* is positive or a sink term if *g* is negative, ${\vartheta }:{\mathbb {R}}\rightarrow {\mathbb {R}}$ denotes the dimensionless active pore volume, and ${\textbf{K}}:{\mathbb {R}}\rightarrow {\mathbb {R}}^{d\times d}$ is the hydraulic conductivity with the physical unit $\textrm{L}\cdot \textrm{T}^{-1}$ (L = length, T = time). Moreover, $u_D$ is a trace of a function $u^*\in L^2(0,T;H^1({\varOmega }))$ on ${\varGamma _\textrm{D}}\times (0,T)$, $g_N\in L^2(0,T; L^2({\varGamma _\textrm{N}}))$ and $u_0\in L^2({\varOmega })$. We note that with respect to (1), we should write ${\vartheta }= {\vartheta }(u-z)$ and ${\textbf{K}}= {\textbf{K}}(\theta (u-z))$, however, we skip this notation for simplicity. We assume that the function ${\vartheta }(u)$ is non-negative, non-decreasing and Lipschitz continuous. Moreover, the tensor ${\textbf{K}}(u)$ is symmetric, positively semi-definite, and Lipschitz continuous.

In order to introduce the weak solution, we set $H(\textrm{div},\varOmega )=\{v\in L^2({\varOmega })^d:\nabla \cdot v\in L^2({\varOmega })\}$ and define the spaces

4 $$\begin{aligned} X&=L^2(0,T,H^1({\varOmega })),\qquad  &   V = \{ v \in X: v|_{{\varGamma _\textrm{D}}} = 0 \}, \\ Y&=\{v\in X: {\vartheta }^\prime (v) \in L^2(0,T,L^2({\varOmega }))\} ,  &   Y^0 =\{v\in Y: v(0)=u_0\},\nonumber \end{aligned}$$

where ${\vartheta }^\prime (u)=\partial _t{\vartheta }(u) = \frac{{\textrm{d}} {\vartheta }}{{\textrm{d}}u} \partial _tu$ denotes the time derivative (in the weak sense). Obviously, if $v\in Y$ then ${\vartheta }(v)\in C([0,T],L^2({\varOmega }))$. In order to shorten the notation, we set the physical flux

5 $$\begin{aligned} {\sigma }(u,\nabla u) := {\textbf{K}}(u)\nabla u,\qquad u\in X. \end{aligned}$$

### Definition 1

We say that $u\in Y$ is the *weak solution* of (3) if $u-u^* \in V$ and

6 $$\begin{aligned} \int ^T_0 \left( \left( {{\vartheta }^\prime (u)},{v}\right) _{\varOmega } + \left( {{\sigma }(u,\nabla u)},{\nabla v}\right) _{\varOmega }-\left( {g},{v}\right) _{\varOmega } -\big ({g_N},{v}\big )_{{\varGamma _\textrm{N}}}\right) {\,{\mathrm d}t}=0\quad \forall v\in V, \end{aligned}$$

where $$\big ({u},{v}\big )_{{\varOmega }}:=\int _{\varOmega }u v {\,{\mathrm d}x}$$ and $$\big ({u},{v}\big )_{{\varGamma _\textrm{N}}}:=\int _{\varGamma _\textrm{N}}u v {\,{\mathrm d}S}$$.

The existence and uniqueness of the Richards equation is studied in [2], see also the later works [3, 28].

## Space-time discretization

We briefly describe the discretization of (6) by the *space-time discontinuous Galerkin* (STDG) method, for more details, see [13, 14]. Let $0=t_0<t_1<\ldots <t_r=T$ be a partition of the time interval (0, *T*) and set $I_m=(t_{m-1},t_m)$ and $\tau _m=t_m-t_{m-1}$. For each $m=0,\dots ,r$, we consider a simplicial mesh ${{\mathcal {T}}_h^m}$ covering ${\overline{{\varOmega }}}$. For simplicity, we assume that ${{\mathcal {T}}_h^m}$, $m=0,\dots ,r$ are conforming, i.e., neighbouring elements share an entire edge or face. However, this assumption can be relaxed by the technique from [12].

For each element $K\in {{\mathcal {T}}_h^m}$, we denote by ${\partial K}$ its boundary, ${n_K}$ its unit outer normal and ${h_K}=\text{ diam }(K)$ its diameter. In order to shorten the notation, we write ${{\partial K}_{\!N}}:={\partial K}\cap {\varGamma _\textrm{N}}$. By the generic symbol $\gamma$, we denote an edge ($d=2$) or a face ($d=3$) of $K\in {{\mathcal {T}}_h^m}$ and $h_{\gamma }$ denotes its diameter. In the following, we speak only about edges, but we mean faces for $d=3$. We assume that

- ${{\mathcal {T}}_h^m}$, $m=0,\dots , r$ are *shape regular*, i.e., ${h_K}/\rho _K\le C$ for all $K\in {{\mathcal {T}}_h}$, where $\rho _K$ is the radius of the largest *d*-dimensional ball inscribed in *K* and constant *C* does not depend on ${{\mathcal {T}}_h^m}$ for $h\in (0,h_0)$, $m=0,\dots ,r$.
- ${{\mathcal {T}}_h^m}$, $m=0,\dots , r$ are *locally quasi-uniform*, i.e., ${h_K}\le C h_{K^\prime }$ for any pair of two neighbouring elements *K* and $K^\prime$, where the constant *C* does not depend on $h\in (0,h_0)$, $m=0,\dots ,r$.

Let $p_K\ge 1$ be an integer denoting the degree of polynomial approximation on $K\in {{\mathcal {T}}_h^m}$, $m=0,\dots ,r$ and $P_{p_K}(K)$ be the corresponding space of polynomial functions on *K*. Let

7 $$\begin{aligned} {S_{hp,m}}=\{v\in L^2({\varOmega }):v|_K\in P_{p_K}(K),\ K\in {{\mathcal {T}}_h^m}\},\qquad m=0,\dots ,r \end{aligned}$$

denote the spaces of discontinuous piecewise polynomial functions on ${{\mathcal {T}}_h^m}$ with possibly varying polynomial approximation degrees. Furthermore, we consider the space of *space-time* discontinuous piecewise polynomial functions

8 $$\begin{aligned} {{S_{hp}^{\tau q}}} =\{v\in L^2({\varOmega }\times (0,T)):\ v|_{I_m}\in P_{q}(I_m,{S_{hp,m}}),\ m=1,\dots , r\}, \end{aligned}$$

where $q\ge 0$ denotes the time polynomial approximation degree and $P_{q}(I_m,{S_{hp,m}})$ is the Bochner space, i.e., $v\in P_{q}(I_m,{S_{hp,m}})$ can be written as $v(x,t)=\sum _{j=0}^q t^j\,v_j(x)$, $v_j\in {S_{hp,m}}$, $j=0,\dots ,q$.

For $v\in {{S_{hp}^{\tau q}}}$, we define the one-sided limits and time jumps by

9 $$\begin{aligned}&v^m_+ =\lim _{t\rightarrow t_m^+}v(t), \ \ m=0,\ldots ,r-1, \qquad v^m_- =\lim _{t\rightarrow t_m^-}v(t), \ \ m=1,\ldots ,r ,\\&\big \{{v}\big \}_m=v^m_+-v^m_-,\quad m=1,\ldots ,r-1,\qquad v^0_-= {\vartheta }(u_0),\qquad \{v\}_0=v^0_+-{\vartheta }(u_0), \nonumber \end{aligned}$$

where $u_0$ is the initial condition. In the following, we use the notation

10 $$\begin{aligned} \big ({u},{v}\big )_{M}&=\int _M u\, v {\,{\mathrm d}x},\qquad \big ({u},{v}\big )_{M,m}=\int _{M\times I_m} u\, v {\,{\mathrm d}x}{\,{\mathrm d}t}, \quad m=1,\dots ,r, \end{aligned}$$

where *M* is either element $K\in {{\mathcal {T}}_h^m}$ or its (part of) boundary ${\partial K}$. The corresponding norms are denoted by ${\left\| \cdot \right\| }_{M}$ and ${\left\| \cdot \right\| }_{M,m}$, respectively. By $\sum _{K,m}=\sum _{m=1}^r\sum _{K\in {{\mathcal {T}}_h^m}}$, we denote the sum over all space-time elements $K\times I_m$, where $K\in {{\mathcal {T}}_h^m}$ and $m=1,\ldots ,r$.

Moreover, we define the jumps and mean values of $v\in {S_{hp,m}}$ on edges $\gamma \subset {\partial K},\ K\in {{\mathcal {T}}_h^m}$ by

11 $$\begin{aligned} [{v}]= {\left\{ \begin{array}{ll} (v^{\scriptscriptstyle (+)} -v^{\scriptscriptstyle (-)} ){n_K}&  \quad \text{ for } \gamma \in {\varOmega }, \\ (v^{\scriptscriptstyle (+)} -u_D){n_K}&  \quad \text{ for } \gamma \subset {\varGamma _\textrm{D}}, \\ 0 &  \quad \text{ for } \gamma \subset {\varGamma _\textrm{N}}, \end{array}\right. } \qquad \left\langle {v}\right\rangle = {\left\{ \begin{array}{ll} (v^{\scriptscriptstyle (+)} +v^{\scriptscriptstyle (-)} )/2 & \quad \text{ for } \gamma \in {\varOmega }, \\ v^{\scriptscriptstyle (+)} & \quad \text{ for } \gamma \subset {\varGamma _\textrm{D}}, \\ 0 & \quad \text{ for } \gamma \subset {\varGamma _\textrm{N}}, \end{array}\right. } \end{aligned}$$

where $v^{\scriptscriptstyle (+)}$ and $v^{\scriptscriptstyle (-)}$ denote the traces of *v* on ${\partial K}$ from interior and exterior of *K*, respectively, and $u_D$ comes from the Dirichlet boundary condition. For vector-valued $v\in [{S_{hp,m}}]^d$, we set $[{v}] = (v^{\scriptscriptstyle (+)} -v^{\scriptscriptstyle (-)} )\cdot {n_K}$ for $\gamma \in {\varOmega }$ and similarly for boundary edges.

For each space-time element $K\times I_m$, $K\in {{\mathcal {T}}_h^m}$, $m=1,\ldots ,r$, we define the forms

12 $$\begin{aligned} {a_{K,m}}(u,v)&:= \big ({{\textbf{K}}(u)\nabla u},{\nabla v}\big )_{K,m} -\big ({g},{v}\big )_{K,m} -\big ({g_N},{v}\big )_{{{\partial K}_{\!N}},m}, \\ {A_{K,m}}(u,v)&:= \big ({{\textbf{K}}(u)\nabla u},{\nabla v}\big )_{K,m} - \big ({\left\langle {{\textbf{K}}(u) \nabla u}\right\rangle \cdot {{n_K}} - \alpha [{u}]\cdot {{n_K}}},{v}\big )_{{{\partial K}\setminus {\varGamma _\textrm{N}}},m} \nonumber \\&\quad + (\beta -\tfrac{1}{2}) \big ({{\textbf{K}}(u)[{u}]},{ \nabla v}\big )_{{{\partial K}\setminus {\varGamma }},m} + (2\beta -1) \big ({{\textbf{K}}(u)[{u}]},{ \nabla v}\big )_{{{\partial K}\cap {\varGamma _\textrm{D}}},m}\nonumber \\&\quad -\big ({g},{v}\big )_{K,m} -\big ({g_N},{v}\big )_{{{\partial K}_{\!N}},m} ,\nonumber \end{aligned}$$

where $\alpha >0$ is a sufficiently large penalization parameter ($\alpha \sim p_K^2/h_K$) and $\beta \in \{0,\tfrac{1}{2},1\}$ corresponds to the choice of the variants of the interior penalty discretization (SIPG with $\beta =0$, IIPG with $\beta =1/2$ and NIPG with $\beta =1$), see, e.g., [13, Chapter 2].

We introduce the space-time discontinuous Galerkin discretization of (3).

### Definition 2

The function ${u_h^{\tau }}\in {{S_{hp}^{\tau q}}}$ is called the approximate solution of (6) obtained by the space-time discontinuous Galerkin method (STDGM), if

13 $$\begin{aligned} \sum _{K,m} {B_{K,m}}({u_h^{\tau }}, v) = 0\qquad \forall v\in {{S_{hp}^{\tau q}}}, \end{aligned}$$

where

14 $$\begin{aligned} {B_{K,m}}(u,v) :=\big ({{\vartheta }^\prime (u)},{v}\big )_{K,m}+{A_{K,m}}\left( u,v \right) +\big ({ \big \{{{\vartheta }(u)}\big \}_{m-1}},{v^{m-1}_+}\big )_{K} \end{aligned}$$

with form ${A_{K,m}}$ given by (12) and $\{\cdot \}$ defined by (9).

### Remark 1

We note that ${u_h^{\tau }}$ is discontinuous with respect to time at $t_m,\ m=1,\dots ,r-1$. The solution between $I_{m-1}$ and $I_m$ is stuck together by the “time-penalty” term $\big ({ \big \{{{\vartheta }(u)}\big \}_{m-1}},{v^{m-1}_+}\big )_{K}$ which also makes sense for *u* and *v* belonging to different finite element spaces.

Finally, we derive some identities that will be used later. Let ${{\mathcal {F}}_h^m}$ denote the set of all interior edges $\gamma \not \subset {\varGamma }$ of mesh ${{\mathcal {T}}_h^m}$ and ${{\mathcal {F}}_D^m}$ the set of boundary edges of ${{\mathcal {T}}_h^m}$ lying on ${\varGamma _\textrm{D}}$. Then, the identity

15 $$\begin{aligned} \sum _{K\in {{\mathcal {T}}_h^m}} \big ({ w},{z\,{n_K}}\big )_{{{\partial K}\setminus {\varGamma _\textrm{N}}},m}= \sum _{\gamma \in {{\mathcal {F}}_h^m}}\left( \big ({\left\langle {w}\right\rangle },{ [{z}]}\big )_{\gamma ,m} +\big ({[{w}] },{ \left\langle {z}\right\rangle }\big )_{\gamma ,m} \right) +\sum _{\gamma \in {{\mathcal {F}}_D^m}}\big ({ w\cdot {n_K}},{z}\big )_{\gamma ,m} \end{aligned}$$

holds for a piecewise smooth vector-valued function *w* and a piecewise smooth scalar function *z*.

Using identity (15) and the following obvious formulas valid for interior edges $\left\langle {\left\langle {{\textbf{K}}(u) \nabla u}\right\rangle }\right\rangle =\left\langle {{\textbf{K}}(u) \nabla u}\right\rangle$, $\left\langle {\alpha [{u}]}\right\rangle =\alpha [{u}]$, $[{\left\langle {{\textbf{K}}(u) \nabla u}\right\rangle }]=0$, $[{\alpha [{u}]}]=0$, we gain

16 $$\begin{aligned} \sum _{K\in {{\mathcal {T}}_h^m}} \big ({\left\langle {{\textbf{K}}(u) \nabla u}\right\rangle \cdot {{n_K}}},{v}\big )_{{{\partial K}\setminus {\varGamma _\textrm{N}}},m}&= \sum _{\gamma \in {{\mathcal {F}}_h^m}} \big ({\left\langle {{\textbf{K}}(u) \nabla u}\right\rangle },{ [{v}] }\big )_{\gamma ,m}+ \sum _{\gamma \in {{\mathcal {F}}_D^m}} \big ({{\textbf{K}}(u) \nabla u \cdot {n_K}},{ v}\big )_{\gamma ,m},\nonumber \\ \sum _{K\in {{\mathcal {T}}_h^m}} \big ({\alpha [{u}]\cdot {{n_K}}},{v}\big )_{{{\partial K}\setminus {\varGamma _\textrm{N}}},m}&= \sum _{\gamma \in {{\mathcal {F}}_h^m}}\big ({\alpha [{u}]},{ [{v}] }\big )_{\gamma ,m}+ \sum _{\gamma \in {{\mathcal {F}}_D^m}} \big ({\alpha [{u}]\cdot {n_K}},{ v}\big )_{\gamma ,m}, \\ \sum _{K\in {{\mathcal {T}}_h^m}}\big ({{\textbf{K}}(u)[{u}]},{ \nabla v}\big )_{{{\partial K}\setminus {\varGamma }},m}&=\sum _{K\in {{\mathcal {T}}_h^m}}\big ({[{u}]},{ {\textbf{K}}(u)\nabla v}\big )_{{{\partial K}\setminus {\varGamma }},m} =2\!\sum _{\gamma \in {{\mathcal {F}}_h^m}}\big ({[{u}] },{ \left\langle {{\textbf{K}}(u)\nabla v}\right\rangle }\big )_{\gamma ,m},\nonumber \\ \sum _{K\in {{\mathcal {T}}_h^m}}\big ({{\textbf{K}}(u)[{u}]},{ \nabla v}\big )_{{{\partial K}\cap {\varGamma _\textrm{D}}},m}&=\sum _{\gamma \in {{\mathcal {F}}_D^m}}\big ({[{u}] },{ {\textbf{K}}(u)\nabla v}\big )_{\gamma ,m}\nonumber . \end{aligned}$$

Consequently, from (12) and (16), we obtain the identity

17 $$\begin{aligned} \sum _{K\in {{\mathcal {T}}_h^m}} {A_{K,m}}(u,v)&=\sum _{K\in {{\mathcal {T}}_h^m}}\big ({{\textbf{K}}(u)\nabla u},{\nabla v}\big )_{K,m}-\sum _{\gamma \in {{\mathcal {F}}_h^m}} \big ({\left\langle {{\textbf{K}}(u) \nabla u}\right\rangle },{ [{v}] }\big )_{\gamma ,m}\\&\quad +(2\beta -1)\sum _{\gamma \in {{\mathcal {F}}_h^m}}\big ({[{u}] },{ \left\langle {{\textbf{K}}(u)\nabla v}\right\rangle }\big )_{\gamma ,m} - \sum _{\gamma \in {{\mathcal {F}}_D^m}} \big ({{\textbf{K}}(u) \nabla u \cdot {n_K}},{ v}\big )_{\gamma ,m} \nonumber \\&\quad + (2\beta -1)\sum _{\gamma \in {{\mathcal {F}}_D^m}}\big ({[{u}] },{ {\textbf{K}}(u)\nabla v}\big )_{\gamma ,m}+\sum _{\gamma \in {{\mathcal {F}}_h^m}}\big ({\alpha [{u}]},{ [{v}] }\big )_{\gamma ,m}\nonumber \\&\quad + \sum _{\gamma \in {{\mathcal {F}}_D^m}} \big ({\alpha [{u}]\cdot {n_K}},{ v}\big )_{\gamma ,m} -\big ({g},{v}\big )_{\varOmega ,m} -\big ({g_N},{v}\big )_{{\varGamma _\textrm{N}},m}. \nonumber \end{aligned}$$

## A Posteriori Error Analysis

### Error Measures

In order to proceed to the derivation of error estimators, we define the spaces of piecewise continuous functions with respect to time by

18 $$\begin{aligned} {Y^\tau }&=\{v\in X: {\vartheta }^\prime (v)|_{I_m}\in L^2(I_m,L^2(\varOmega ))\},\qquad {V^\tau }=\{v\in {Y^\tau }: v|_{{\varGamma _\textrm{D}}\times (0,T)} = 0\}. \end{aligned}$$

Obviously, $Y^0\subset Y\subset {Y^\tau }\subset X$ and ${{S_{hp}^{\tau q}}} \subset {Y^\tau }$. Moreover, we have the following result.

#### Lemma 1

Let $u\in Y^0$ be the weak solution of (6). Then it satisfies

19 $$\begin{aligned} \sum _{K,m}{b_{K,m}}(u,v)&=0\quad \forall v\in {V^\tau }, \end{aligned}$$

where

20 $$\begin{aligned} {b_{K,m}}(u,v) :=\big ({{\vartheta }^\prime (u)},{v}\big )_{K,m}+{a_{K,m}}(u,v) +\big ({\{{\vartheta }(u)\}_{m-1}},{v^{m-1}_+}\big )_{K} \end{aligned}$$

with ${a_{K,m}}$ given by (12) and the time jump $\{\cdot \}_{m-1}$ defined by (9). Moreover, there exists a unique solution $u\in Y^\tau$ such that $u-u^*\in V^\tau$ and satisfies (19).

#### Proof

The proof follows directly by comparing formulas (19)–(20) with (6) and the fact that $\big ({\{{\vartheta }(u)\}_{m-1}},{v^{m-1}_+}\big )_{K}=0$ for $u\in Y^0$. For the proof of uniqueness, we employ the fact that $C_0^\infty (\varOmega )$ is dense in $L^2(\varOmega )$, i.e., there exists a sequence $\{v_\varepsilon \}\subset C_0^\infty (\varOmega )$ for any $v\in L^2(\varOmega )$ such that $\Vert v_\varepsilon -v\Vert \rightarrow 0$ as $\varepsilon \rightarrow 0$, cf. [34, Theorem 3.14]. We apply $v=v_{s,\varepsilon _1}(x)v_{t,\varepsilon _2}(t)$ in (19), where the spatial component $v_{s,\varepsilon _1}\in \{v\in H^1(\varOmega ):v|_{{\varGamma _\textrm{D}}}=0\}$ tends to $\{{\vartheta }(u)\}_{m-1}$ as $\varepsilon _1\rightarrow 0$ and the time component $v_{t,\varepsilon _2}$ is given as 0 outside the interval $(t_{m-1},t_{m-1}+\varepsilon _2)$ and $v_{t,\varepsilon _2}=1-(t-t_{m-1})/\varepsilon _2$ on $(t_{m-1},t_{m-1}+\varepsilon _2)$, i.e., $v_{t,\varepsilon _2}(t)$ tends to 0 as $\varepsilon _2\rightarrow 0$. Therefore, all the terms containing time integrals in (19) tend to 0 when $\varepsilon _2$ tends to 0. Since $v^{m-1}_+=v_{s,\varepsilon _1}$, the remaining jump term tends to $\Vert \{{\vartheta }(u)\}_{m-1}\Vert ^2$ as $\varepsilon _1$ tends to 0. From this it follows that $\{{\vartheta }(u)\}_{m-1}=0$. Then it is possible to see that any solution of (19) satisfies the original weak formulation (6). Since the weak problem (6) has a unique solution, cf. [2], the extended problem (19) has a unique solution as well. $\square$

In virtue of [11, § 2.3.1], we define a parameter ${d_{K,m}}$ associated with the space-time element $K\times I_m$, $K\in {{\mathcal {T}}_h^m}$, $m=1,\dots ,r$. The parameter ${d_{K,m}}$ represents a user-dependent weight, typically with physical units $(\textrm{T}\,\textrm{L})^{1/2}$ so that the error measure has the same physical unit as the energy norm. In this paper, we use two choices

21a $$\begin{aligned} {d_{K,m}}&:= \left( h_K^{-2} {\left\| {\textbf{K}}(u_h)\right\| }_{m,\infty } + \tau _m^{-2} T {\left\| \tfrac{{\mathrm d}{\vartheta }}{{\mathrm d}u}(u_h)\right\| }_{m,\infty } \right) ^{-1/2}, \end{aligned}$$

21b $$\begin{aligned} {d_{K,m}}&:= \left( {h_K^{2}}{{\left\| {\textbf{K}}(u_h)\right\| }_{m,\infty }^{-1} } + {\tau _m^{2}}{ /T {\left\| \tfrac{{\mathrm d}{\vartheta }}{{\mathrm d}u}(u_h)\right\| }_{m,\infty }^{-1} } \right) ^{1/2}. \end{aligned}$$

where ${\left\| \cdot \right\| }_{m,\infty } := {\left\| \cdot \right\| }_{L^\infty ({\varOmega }\times I_m)}$. We note that the following error analysis is independent of the choice of ${d_{K,m}}$. Moreover, we define the norm in the space ${V^\tau }$ (cf. (18)) by

22 $$\begin{aligned} {\left\| v\right\| }_{{V^\tau }}^{2} =\sum _{K,m} {\left\| v\right\| }_{V_{K,m}}^{2} , \quad {\left\| v\right\| }_{V_{K,m}}^{2} = {{d_{K,m}^{-2}}} \left( h_K^2{\left\| \nabla v\right\| }_{K,m}^{2} +\tau _m^2 {\left\| v^{\prime }\right\| }_{K,m}^{2} \right) . \end{aligned}$$

In virtue of (19), we introduce the error measure as a dual norm of the residual

23 $$\begin{aligned} {{\mathcal {R}}}({u_h^{\tau }})=\sup _{0\ne v\in {V^\tau }}\frac{\sum _{K,m}{b_{K,m}}({u_h^{\tau }},v)}{{\left\| v\right\| }_{{V^\tau }} }, \end{aligned}$$

where ${b_{K,m}}$ is given by (20). The residual ${{\mathcal {R}}}(v)$ represents a natural error measure for $u-v\in {V^\tau }$, cf. [11, Remark 2.3]. In Sect. 4, we estimate ${{\mathcal {R}}}({u_h^{\tau }})$ for ${u_h^{\tau }}$ being the solution of (13).

Since the approximate solution ${u_h^{\tau }}$ belongs to the space of discontinuous function ${{S_{hp}^{\tau q}}}\not \subset {V^\tau }$, we introduce the second building block measuring the nonconformity of the solution in spatial variables. Therefore, similarly to [18], we define the form

24 $$\begin{aligned} {{\mathcal {J}}}(v)=\sum _{K,m}{{J_{K,m}}}(v),\quad {{J_{K,m}}}(v)={{d_{K,m}^{2}}}\,\tau _m^{-1}\, {h_{K}^{-2}}\,C_{K,m,{\textbf{K}},\alpha }{\left\| [v]\right\| }_{{\partial K},m}^{2} , \end{aligned}$$

where $C_{K,m,{\textbf{K}},\alpha }=\alpha ^2 +{\left\| {\textbf{K}}({u_h^{\tau }})\right\| }_{L^\infty (K\times I_m)}^{2}$. The scaling factors are chosen such that ${{\mathcal {J}}}(v)^{1/2}$ has the same physical unit as ${{\mathcal {R}}}({u_h^{\tau }})$.

We note that ${{\mathcal {J}}}(v)$ measures also the violation of the Dirichlet boundary condition since ${{\mathcal {J}}}(v)$ contains the term ${\left\| v-u_D\right\| }_{{\partial K}\cap {\varGamma _\textrm{D}},m}$, cf. (11).

The final error measure is then defined by

25 $$\begin{aligned} {{\mathcal {E}}}({u_h^{\tau }}): =\left( {{\mathcal {R}}}({u_h^{\tau }})^2+{{\mathcal {J}}}({u_h^{\tau }})\right) ^{1/2}, \end{aligned}$$

where ${{\mathcal {R}}}({u_h^{\tau }})$ is given by (23) and ${{\mathcal {J}}}({u_h^{\tau }})$ by (24).

#### Lemma 2

The error measure ${{\mathcal {E}}}({u_h^{\tau }})=0$ if and only if ${u_h^{\tau }}=u$ is the weak solution given by (6).

#### Proof

Obviously, if ${u_h^{\tau }}=u$, then ${{\mathcal {J}}}({u_h^{\tau }})=0$ and ${{\mathcal {R}}}({u_h^{\tau }})=0$ due to (19). On the other hand, if ${{\mathcal {J}}}({u_h^{\tau }})=0$, then ${u_h^{\tau }}\in {Y^\tau }$ and ${u_h^{\tau }}-u^*\in {V^\tau }$. Moreover, ${{\mathcal {R}}}({u_h^{\tau }})=0$ and the uniqueness of (19) imply that ${u_h^{\tau }}$ is the weak solution (6). $\square$

### Temporal and Spatial Flux Reconstructions

Similarly as in [18], we define a temporal reconstruction ${R_h^{\tau }} = {R_h^{\tau }}(x,t)$ as a continuous function with respect to time that mimics ${\vartheta }({u_h^{\tau }})$, ${u_h^{\tau }}\in {{S_{hp}^{\tau q}}}$. Let $r_m\in P_{q+1}(I_m)$ be the right Radau polynomial on $I_m$, i.e., $r_m(t_{m-1})=1$ and $r_m(t_m)=0$, and $r_m$ is orthogonal to $P_{q-1}(I_m)$ with respect to the $L^2(I_m)$ inner product. Then we set

26 $$\begin{aligned} {R_h^{\tau }}(x,t):={\vartheta }({u_h^{\tau }}(x,t))- \big \{{{\vartheta }({u_h^{\tau }})}\big \}_{m-1}(x)\,r_m(t), \quad x\in \varOmega ,\, t\in I_m, \end{aligned}$$

where $\big \{{\cdot }\big \}$ is given by (9). The *temporal flux reconstruction* ${R_h^{\tau }}(x,t)$ is continuous in time, namely ${R_h^{\tau }}\in H^1(0,T, L^2({\varOmega }))$ and it satisfies the initial condition due to

27 $$\begin{aligned} {R_h^{\tau }}(\cdot ,0)&= {\vartheta }({u_h^{\tau }}(\cdot ,0)) - \{ {\vartheta }({u_h^{\tau }}) \}_0(\cdot ) r_1(0) \\&= {\vartheta }({u_h^{\tau }}(\cdot ,0)) - ({\vartheta }({u_h^{\tau }}(\cdot ,0)) - {\vartheta }(u_0(\cdot )) = {\vartheta }(u_0(\cdot )). \nonumber \end{aligned}$$

Moreover, by the integration by parts and the properties $r_m(t_{m-1})=1$, $r_m(t_m)=0$, we obtain

28 $$\begin{aligned} \big ({({R_h^{\tau }}- {\vartheta }({u_h^{\tau }}))^\prime },{v}\big )_{K,m}&= - \big ({r^\prime _m \big \{{{\vartheta }({u_h^{\tau }})}\big \}_{m-1}},{ v}\big )_{K,m} \\&= \big ({r_m \big \{{{\vartheta }({u_h^{\tau }})}\big \}_{m-1}},{v^\prime }\big )_{K,m} - r_m(t_m)\big ({ \big \{{{\vartheta }({u_h^{\tau }})}\big \}_{m-1}},{ v^m_{-}}\big )_{K}\nonumber \\&\quad + r_m(t_{m-1})\big ({ \big \{{{\vartheta }({u_h^{\tau }})}\big \}_{m-1}},{ v^{m-1}_{+}}\big )_{K} \nonumber \\&= \big ({r_m \big \{{{\vartheta }({u_h^{\tau }})}\big \}_{m-1}},{ v^\prime }\big )_{K,m} + \big ({ \big \{{{\vartheta }({u_h^{\tau }})}\big \}_{m-1}},{ v^{m-1}_+}\big )_{K}, \qquad v \in {V^\tau }, \nonumber \end{aligned}$$

which together with definition (26) implies

29 $$\begin{aligned} \big ({ ({R_h^{\tau }}- {\vartheta }({u_h^{\tau }}))^\prime },{ v}\big )_{K,m} - \big ({ \big \{{{\vartheta }({u_h^{\tau }})}\big \}_{m-1}},{ v^{m-1}_+}\big )_{K} = - \big ({{R_h^{\tau }}- {\vartheta }({u_h^{\tau }})},{v^\prime }\big )_{K,m}, \quad v\in {V^\tau }. \end{aligned}$$

Finally, using the orthogonality of $r_m$ to $P_{q-1}(I_m)$, we obtain from (28), the formula

30 $$\begin{aligned} \left( {({R_h^{\tau }}-{\vartheta }({u_h^{\tau }}))^\prime },{v}\right) _{m,K} = \left( {\{ {\vartheta }({u_h^{\tau }}) \}_{m-1}},{v^{m-1}_+}\right) _{K} \quad \forall v\in P_q(I_m,L^2(K)). \end{aligned}$$

Consequently, if ${u_h^{\tau }}$ is the approximate solution given by (13), then it satisfies

31 $$\begin{aligned} \big ({({R_h^{\tau }})^\prime },{v}\big )_{K,m}&=\big ({{\vartheta }^{\prime }({u_h^{\tau }})},{v}\big )_{K,m} + \big ({ \big \{{{\vartheta }({u_h^{\tau }})}\big \}_{m-1}},{v^{m-1}_+}\big )_{K}=-{A_{K,m}}({u_h^{\tau }},v) \\&\quad \forall v\in P_q(I_m,P_{p_K}(K)).\nonumber \end{aligned}$$

Obviously, the reconstruction ${R_h^{\tau }}$ is local and explicit, so its computation is fast and easy to implement.

The *spatial flux reconstruction* needs to define a function ${\sigma _h^{\tau }}\in L^2(0,T,H(\textrm{div},\varOmega ))$ which mimics the flux ${\sigma }({u_h^{\tau }}, \nabla {u_h^{\tau }}) = {\textbf{K}}({u_h^{\tau }})\nabla {u_h^{\tau }}$, cf. (5). In particular, ${\sigma _h^{\tau }}|_{K\times I_m} \in P_q(I_m, {\textrm{RTN}}_{p}(K))$ where

32 $$\begin{aligned} {\textrm{RTN}}_{p}(K)=P_{p}(K)^d+x\,P_{p}(K),\qquad K\in {{\mathcal {T}}_h}, \ m=1,\dots ,r \end{aligned}$$

is the Raviart-Thomas-Nedelec finite elements, cf. [7] for more details. We assume that the reconstructed flux ${\sigma _h^{\tau }}$ has to be equilibrated with the temporal flux ${R_h^{\tau }}$

33 $$\begin{aligned} \big ({\nabla \cdot {{\sigma _h^{\tau }}}},{v}\big )_{K,m} = \big ({({R_h^{\tau }})^{\prime } - g},{v}\big )_{K,m} \quad \forall v\in P_q(I_m,P_{p_K}(K)),\ K\in {{\mathcal {T}}_h^m}, \end{aligned}$$

and with the Neumann boundary condition

34 $$\begin{aligned} \big ({{{\sigma _h^{\tau }}}\cdot n},{v}\big )_{\gamma ,m} = \big ({g_N},{v}\big )_{\gamma ,m} \quad \forall v\in P_q(I_m,P_{p_K}(\gamma ))\ \forall \gamma \subset {{\partial K}_{\!N}},\ K\in {{\mathcal {T}}_h^m}. \end{aligned}$$

In Sect. 5 we present two possible constructions of ${\sigma _h^{\tau }}$ including the choice of the spatial polynomial degree *p* in (32).

### Auxiliary Results

In the forthcoming numerical analysis, we need several technical tools. We will employ the *scaled space-time Poincarè inequality*, cf. [11, Lemma 2.2]: Let $\varphi _{K,m}\in P_0(K\times I_m)$ be the $L^2$-orthogonal projection of $\varphi \in H^1(K\times I_m)$ onto a constant in each space-time element $K \times I_m$, $K\in {{\mathcal {T}}_h^m}$, $m=0,\dots ,r$. Then,

35 $$\begin{aligned} {\left\| \varphi - \varphi _{K,m}\right\| }_{K,m} \le C_{\textrm{P}}\left( {h_K^2}{\left\| \nabla \varphi \right\| }_{K,m}^{2} + \tau ^2_m {\left\| \varphi ^\prime \right\| }_{K,m}^{2} \right) ^{1/2} = C_{\textrm{P}}{d_{K,m}}{\left\| \varphi \right\| }_{V_{K,m}} , \end{aligned}$$

where $C_{\textrm{P}}$ is the Poincarè constant equal to $1/\pi$ for simplicial elements and the last equality follows from (22).

Moreover, we introduce the *space-time trace inequality*

#### Lemma 3

Let $\varphi _{\gamma ,m}\in P_0(\gamma \times I_m)$ be the $L^2$-orthogonal projection of $\varphi \in H^1(K\times I_m)$ onto a constant on each $\gamma \times I_m$, where $\gamma \subset {\partial K}$ is an edge of $K\in {{\mathcal {T}}_h^m}$. Then there exists a constant $C_{\textrm{T}}>0$ such that

36 $$\begin{aligned} {\left\| \varphi - \varphi _{\gamma ,m}\right\| }_{\gamma \times I_m} \le C_{\textrm{T}}\max (1,h_{\gamma }^{-1/2}) {d_{K,m}}{\left\| \varphi \right\| }_{V_{K,m}} , \end{aligned}$$

where $C_{\textrm{T}}= \max (c_T,C_{\textrm{P}})$, $C_{\textrm{P}}$ is from (35) and $c_T>0$ is the constant from the (space) trace inequality.

#### Proof

The proof is straightforward, we present it for completeness. Let $\varphi \in H^1(K \times I_m)$ and, for all $t\in I_m$, set ${{\tilde{\varphi }}}(t):= |\gamma |^{-1} \int _{\gamma } \varphi (x,t){\,{\mathrm d}S}$. Observing that $(\varphi -{{\tilde{\varphi }}})$ and $({{\tilde{\varphi }}}-\varphi _{\gamma ,m})$ are $L^2(\gamma \times I_m)$-orthogonal, we have

37 $$\begin{aligned} {\left\| \varphi - \varphi _{\gamma ,m}\right\| }_{\gamma \times I_m}^{2} = {\left\| \varphi - {{\tilde{\varphi }}}\right\| }_{\gamma \times I_m}^{2} + {\left\| {{\tilde{\varphi }}} - \varphi _{\gamma ,m}\right\| }_{\gamma \times I_m}^{2} . \end{aligned}$$

Using the standard trace inequality (e.g., [21, Lemma 3.32]), we have

38 $$\begin{aligned} {\left\| \varphi (\cdot , t) - {\tilde{\varphi }}(t)\right\| }_{\gamma } \le c_T h_{\gamma }^{1/2} {\left\| \nabla \varphi \right\| }_{K} \qquad \forall t\in I_m, \end{aligned}$$

where $c_T>0$ is a constant whose values can be set relatively precisely, see the discussion in [37, Section 4.6]. Hence, integrating the square of (38) over $I_m$ and using the fact that $h_{\gamma }\le {h_K}$, $\gamma \subset {h_K}$, we estimate the first term on the right-hand side of (37) as

39 $$\begin{aligned} {\left\| \varphi - {\tilde{\varphi }}\right\| }_{\gamma \times I_m}^{2} \le c_T^2 h_{\gamma }{\left\| \nabla \varphi \right\| }_{K\times I_m}^{2} \le c_T^2 h_{\gamma }^{-1} {h_K^2}{\left\| \nabla \varphi \right\| }_{K\times I_m}^{2} . \end{aligned}$$

Using the fact that $\varphi _{\gamma ,m} = \tau _m^{-1} \int _{I_m} {{\tilde{\varphi }}}(t){\,{\mathrm d}t}$, the one-dimensional Poincarè inequality on $I_n$ and the Cauchy–Schwarz inequality yield

40 $$\begin{aligned} {\left\| {{\tilde{\varphi }}} - \varphi _{\gamma ,m}\right\| }_{\gamma \times I_m}^{2}&= |\gamma | \int _{I_m} |{{\tilde{\varphi }}} - \varphi _{\gamma ,m}|^2(t){\,{\mathrm d}t}\le |\gamma | C_{\textrm{P}}^2\tau _m^2 \int _{I_m} |\tfrac{{\mathrm d}}{{\mathrm d}t}{{\tilde{\varphi }}}(t)|^2{\,{\mathrm d}t}\\&= \frac{C_{\textrm{P}}^2\tau _m^2}{|\gamma |} \int _{I_m} \left( \int _\gamma \partial _t\varphi (x,t){\,{\mathrm d}x}\right) ^2 {\,{\mathrm d}t}\le C_{\textrm{P}}^2 \tau _m^2 \int _{I_m} \left( \int _\gamma |\partial _t\varphi |^2{\,{\mathrm d}x}\right) {\,{\mathrm d}t}\nonumber \\&= C_{\textrm{P}}^2 \tau _m^2{\left\| \partial _t\varphi \right\| }_{\gamma \times I_m}^{2} . \nonumber \end{aligned}$$

Collecting bounds (37), (39), (40) and the definition of the norm (22) yields (36). $\square$

### Reliability

We presented the upper bound of ${{\mathcal {R}}}({u_h^{\tau }})$, cf. (23).

#### Theorem 1

Let $u\in Y$ be the weak solution of (6) and ${u_h^{\tau }}\in {{S_{hp}^{\tau q}}}$ be the approximate solution given by (13). Let ${R_h^{\tau }}\in H^1(0,T, L^2({\varOmega }))$ be the temporal reconstruction given by (26) and ${{\sigma _h^{\tau }}}\in L^2(0,T,H(\textrm{div},\varOmega ))$ be the spatial reconstruction satisfying (33). Then

41 $$\begin{aligned} {{\mathcal {R}}}({u_h^{\tau }})^2&\le \eta ^2 := \sum _{K,m} \eta _{K,m}^2,\quad \eta _{K,m}:= C_{\textrm{P}}\eta _{R,K,m}+ (\eta _{S,K,m}^2 + \eta _{T,K,m}^2)^{1/2}+C_{\textrm{T}}\eta _{N,K,m}, \end{aligned}$$

where $C_{\textrm{P}}$ is the constant from Poincarè inequality (35), $C_{\textrm{T}}$ is the constant from the trace inequality (36) and the estimators $\eta _{R,K,m}$, $\eta _{S,K,m}$, $\eta _{T,K,m}$, and $\eta _{N,K,m}$ are given by

42a $$\begin{aligned} \eta _{R,K,m}&:= {d_{K,m}}{\left\| ({R_h^{\tau }})^\prime - \nabla \cdot {\sigma _h^{\tau }}- g \right\| }_{K,m} , \end{aligned}$$

42b $$\begin{aligned} \eta _{S,K,m}&:= \frac{{d_{K,m}}}{{h_K}} {\left\| {\sigma _h^{\tau }}- {\sigma }({u_h^{\tau }}, \nabla {u_h^{\tau }}) \right\| }_{K,m} , \end{aligned}$$

42c $$\begin{aligned} \eta _{T,K,m}&:= \frac{{d_{K,m}}}{\tau _m} {\left\| {R_h^{\tau }}- {\vartheta }({u_h^{\tau }}) \right\| }_{K,m} , \end{aligned}$$

42d $$\begin{aligned} \eta _{N,K,m}&:= \sum _{\gamma \subset {{\partial K}_{\!N}}} \max (1,h_{\gamma }^{-1/2}) {d_{K,m}}{\left\| {\sigma _h^{\tau }}\cdot n- g_N \right\| }_{{{\partial K}_{\!N}},m} . \end{aligned}$$

The proof of Theorem 1 can be found in [19] for the case of the homogeneous Dirichlet boundary condition. For completeness, we present its modification including mixed Dirichlet-Neumann boundary conditions.

#### Proof

Starting from (20), adding the terms $\pm \big ({{R_h^{\tau }}},{v}\big )_{K,m}$ and $\pm \big ({\nabla \cdot {\sigma _h^{\tau }}},{v}\big )_{K,m}$, and using the integration by parts, we obtain

43 $$\begin{aligned}&\sum _{K,m} {b_{K,m}}({u_h^{\tau }}, v) \\&\quad =\sum _{K,m} \left\{ \big ({{\vartheta }^\prime ({u_h^{\tau }})-g},{v}\big )_{K,m} -\big ({g_N},{v}\big )_{{{\partial K}_{\!N}},m} + \big ({{\sigma }({u_h^{\tau }}, \nabla {u_h^{\tau }})},{\nabla v}\big )_{K,m} + \big ({ \big \{{{\vartheta }({u_h^{\tau }})}\big \}_{m-1}},{v^{m-1}_{+}}\big )_{K} \right\} \nonumber \\&\quad =\sum _{K,m} \big ({({R_h^{\tau }})^\prime - \nabla \cdot {\sigma _h^{\tau }}- g},{v}\big )_{K,m} - \sum _{K,m} \big ({{\sigma _h^{\tau }}- {\sigma }({u_h^{\tau }}, \nabla {u_h^{\tau }})},{\nabla v}\big )_{K,m} \nonumber \\&\qquad - \sum _{K,m} \left\{ \big ({({R_h^{\tau }}- {\vartheta }({u_h^{\tau }}))^\prime },{v}\big )_{K,m} - \big ({ \big \{{{\vartheta }({u_h^{\tau }})}\big \}_{m-1}},{v^{m-1}_{+}}\big )_{K} \right\} + \sum _{K,m} \big ({{\sigma _h^{\tau }}\cdot n- g_N},{v}\big )_{{{\partial K}_{\!N}},m} \nonumber \\&\quad =: \xi _1 + \xi _2 + \xi _3 + \xi _4. \nonumber \end{aligned}$$

The terms $\xi _i$, $i=1,\dots ,4$ are estimated separately.

Let ${v_{K,m}}\in P_0(K\times I_m)$ be the piecewise constant projection of $v\in {V^\tau }$ given by the identity $\big ({{v_{K,m}}},{1}\big )_{K,m} = \big ({v},{1}\big )_{K,m}$. Using the Cauchy–Schwarz inequality, assumption (33), the Poincarè inequality (35), and (22), we have

44 $$\begin{aligned} |\xi _1|&\le \sum _{K,m} \big | \big ({({R_h^{\tau }})^\prime - \nabla \cdot {\sigma _h^{\tau }}- g},{v}\big )_{K,m} \big | = \sum _{K,m} \big | \big ({({R_h^{\tau }})^\prime - \nabla \cdot {\sigma _h^{\tau }}- g},{ v - {v_{K,m}}}\big )_{K,m} \big | \\&\le \sum _{K,m} C_{\textrm{P}}{\left\| ({R_h^{\tau }})^\prime - \nabla \cdot {\sigma _h^{\tau }}- g\right\| }_{K,m} \left( {h_K^2}{\left\| \nabla v\right\| }_{K,m}^{2} + \tau ^2_m {\left\| v^\prime \right\| }_{K,m}^{2} \right) ^{1/2} \nonumber \\&= \sum _{K,m} C_{\textrm{P}}\,{d_{K,m}}\,{\left\| ({R_h^{\tau }})^\prime - \nabla \cdot {\sigma _h^{\tau }}- g\right\| }_{K,m} {\left\| v\right\| }_{V_{K,m}} = \sum _{K,m} C_{\textrm{P}}\eta _{R,K,m}{\left\| v\right\| }_{V_{K,m}} . \nonumber \end{aligned}$$

Furthermore, by the Cauchy–Schwarz inequality and (22), we obtain

45 $$\begin{aligned} |\xi _2|&\le \sum _{K,m} \big | \big ({{\sigma _h^{\tau }}- {\sigma }({u_h^{\tau }}, \nabla {u_h^{\tau }})},{\nabla v}\big )_{K,m} \big |\\&\le \sum _{K,m} \frac{{d_{K,m}}}{{h_K}} {\left\| {\sigma _h^{\tau }}- {\sigma }({u_h^{\tau }}, \nabla {u_h^{\tau }})\right\| }_{K,m} \, \frac{{h_K}}{{d_{K,m}}} {\left\| \nabla v\right\| }_{K,m} = \sum _{K,m} \eta _{S,K,m}\frac{{h_K}}{{d_{K,m}}}{\left\| \nabla v\right\| }_{K,m} . \nonumber \end{aligned}$$

The use of (29), and a similar manipulations as in (45), give

46 $$\begin{aligned} |\xi _3|&\le \sum _{K,m} \big | \big ({({R_h^{\tau }}- {\vartheta }({u_h^{\tau }}))^\prime },{v}\big )_{K,m} - \big ({ \big \{{{\vartheta }({u_h^{\tau }})}\big \}_{m-1}},{v^{m-1}_{+}}\big )_{K} \big | = \sum _{K,m} \big | \big ({{R_h^{\tau }}- {\vartheta }({u_h^{\tau }})},{v^\prime }\big )_{K,m} \big |\nonumber \\&\le \sum _{K,m} \frac{{d_{K,m}}}{\tau _m} {\left\| {R_h^{\tau }}- {\vartheta }({u_h^{\tau }})\right\| }_{K,m} \frac{\tau _m}{{d_{K,m}}}{\left\| v^\prime \right\| }_{K,m} = \sum _{K,m} \eta _{T,K,m}\frac{\tau _m}{{d_{K,m}}}{\left\| v^\prime \right\| }_{K,m} . \end{aligned}$$

Hence, estimates (45)–(46), the Cauchy inequality and (22) imply

47 $$\begin{aligned} |\xi _2| + |\xi _3|&\le \sum _{K,m}\left( \eta _{S,K,m}\frac{{h_K}}{{d_{K,m}}}{\left\| \nabla v\right\| }_{K,m} + \eta _{T,K,m}\frac{\tau _m}{{d_{K,m}}}{\left\| v^\prime \right\| }_{K,m} \right) \\&\le \sum _{K,m}\left( \eta _{S,K,m}^2+\eta _{T,K,m}^2\right) ^{1/2}{\left\| v\right\| }_{V_{K,m}} . \nonumber \end{aligned}$$

Furthermore, let ${v_{\gamma ,m}}\in P_0(\gamma \times I_m)$, $\gamma \subset {{\partial K}_{\!N}}$ be the $L^2$-orthogonal projection from Lemma 3. Then using assumption (34), the Cauchy inequality and the space-time trace inequality (36), we have

48 $$\begin{aligned} |\xi _4|&= \sum _{K,m}\sum _{\gamma \subset {{\partial K}_{\!N}}} \big ({{\sigma _h^{\tau }}\cdot n- g_N},{v-{v_{\gamma ,m}}}\big )_{\gamma ,m} \le \sum _{K,m}\sum _{\gamma \subset {{\partial K}_{\!N}}} {\left\| {\sigma _h^{\tau }}\cdot n- g_N\right\| }_{\gamma ,m} {\left\| v-{v_{\gamma ,m}}\right\| }_{\gamma ,m} \nonumber \\&\le C_{\textrm{T}}\sum _{K,m} \sum _{\gamma \subset {{\partial K}_{\!N}}}\max (1,h_{\gamma }^{-1/2}) {d_{K,m}}{\left\| {\sigma _h^{\tau }}\cdot n- g_N\right\| }_{\gamma ,m} {\left\| v\right\| }_{V_{K,m}} . \end{aligned}$$

The particular estimates (44), (47), and (48), together with the discrete Cauchy–Schwarz inequality, imply (41). $\square$

#### Remark 2

Obviously, if ${\partial K}\cap {\varGamma _\textrm{N}}\ne \emptyset$, then $\eta _{N,K,m}=0$.

### Efficiency

The aim is to show that the local individual error estimators $\eta _{R,K,m}$, $\eta _{S,K,m}$ and $\eta _{T,K,m}$ from (41)–(42) are locally efficient, i.e., they provide local lower bounds to the error measure up to a generic constant $C>0$ which is independent of *u*, ${u_h^{\tau }}$, *h* and $\tau$, but may depend on data problems and the degrees of polynomial approximation *p* and *q*. A dependence of the estimate up to this generic constant we will denote by $\lesssim$.

In order to derive the local variants of the error measure, we denote by ${\omega _K}$ the set of elements sharing at least a vertex with $K\in {{\mathcal {T}}_h^m}$, i.e.,

49 $$\begin{aligned} {\omega _K}= \cup _{K^\prime \cap K \ne 0} K^\prime ,\qquad K\in {{\mathcal {T}}_h^m},\ m=0,\dots ,r. \end{aligned}$$

Moreover, we define the functional sub-spaces $V_{D,m} = \{ v \in {V^\tau }: \text{ supp }\,(v) \subset \overline{ D \times I_m } \}$ for any set $D\subset {\varOmega }$ (cf. (18)) and the corresponding error measures (cf. (23))

50 $$\begin{aligned} {{\mathcal {R}}}_{D,m}(w) = \sup _{ \{0 \ne v \in V_{D,m} \} } \frac{1}{ {\left\| v\right\| }_{{V^\tau }} } \sum _{K,m} {b_{K,m}}(w,v). \end{aligned}$$

Obviously, the definition of $V_{D,m}$ and ${{\mathcal {R}}}_{D,m}({u_h^{\tau }})$ together with the shape regularity implies

51 $$\begin{aligned} \sum _{K,m}{{\mathcal {R}}}_{K,m}({u_h^{\tau }}) \le \sum _{K,m}{{\mathcal {R}}}_{{\omega _K},m}({u_h^{\tau }}) \lesssim {{\mathcal {R}}}({u_h^{\tau }}). \end{aligned}$$

Moreover, for each space-time element $K\times I_m$, $K\in {{\mathcal {T}}_h^m}$, $m=1,\dots ,r$, we introduce the $L^2(K\times I_m)$-projection of the non-polynomial functions, namely

52 $$\begin{aligned}&\overline{\vartheta ^\prime ({u_h^{\tau }})}\in P_q(I_m, P_{p_K}(K):\quad \big ({\overline{\vartheta ^\prime ({u_h^{\tau }})}},{v}\big )_{K,m} = \big ({{\vartheta }^\prime ({u_h^{\tau }})},{v}\big )_{K,m} \quad \forall v\in P_q(I_m, P_{p_K}(K)) \nonumber \\&{\overline{g}}\in P_q(I_m, P_{p_K}(K)): \quad \big ({{\overline{g}}},{v}\big )_{K,m} = \big ({g},{v}\big )_{K,m} \quad \forall v \in P_q(I_m, P_{p_K}(K)). \end{aligned}$$

Finally, for each vertex *a* of the mesh ${{\mathcal {T}}_h^m}$, we denote by ${\omega _a}$ a patch of elements $K\in {{\mathcal {T}}_h^m}$ that share this vertex. By $p_a= \max _{K\in {\omega _a}} p_K$ we denote the maximal polynomial degree on ${\omega _a}$. Then, for each *a* of $K\in {{\mathcal {T}}_h^m}$, we define a vector-valued function ${\overline{\sigma }}_a = {\overline{\sigma }}_a({u_h^{\tau }}, \nabla {u_h^{\tau }}) \in P_q(I_m, {\textrm{RTN}}_{p_a}(K))$ (cf. (32)) by

53 $$\begin{aligned} \big ({{\overline{\sigma }}_a \cdot {{n_K}}},{v}\big )_{\gamma ,m}&= \big ({\psi _a \left\langle {{\sigma }({u_h^{\tau }}, \nabla {u_h^{\tau }})}\right\rangle \cdot {n_K}},{v}\big )_{\gamma ,m}\quad \forall v \in P_q(I_m, P_{p_a}(\gamma )),\ \gamma \subset K \\ ({\overline{\sigma }}_a \cdot v)_{K,m}&= (\psi _a {\sigma }({u_h^{\tau }}, \nabla {u_h^{\tau }}) , v)_{K,m}\quad \forall v \in P_q(I_m, P_{p_a-1}(K)^d), \nonumber \end{aligned}$$

where $\left\langle {\cdot }\right\rangle$ denotes the mean value on $\gamma \subset {\partial K}$ and $\psi _a$ is a continuous piecewise linear function such that $\psi _a(a)=1$ and it vanishes at the other vertices of *K*. Finally, we set ${\overline{\sigma }}|_{K\times I_m}=\sum _{{a\in K}}{\overline{\sigma }}_a$.

The proof of the local efficiency of the error estimates presented is based on the choice of a suitable test function in (23). We set

54 $$\begin{aligned} w(x,t) = \frac{{d_{K,m}^{2}}}{\tau _m} P_h\big (\big \{{{\vartheta }({u_h^{\tau }})}\big \}_{m-1}\big )(x) \chi _K(x) \varPhi _m(t). \end{aligned}$$

where $\chi _K(x)$ is the standard bubble function on *K*, $\varPhi _m(t)$ is the Legendre polynomial of degree $q+1$ on $I_m$ (and vanishing outside) and $P_h\big (\big \{{{\vartheta }({u_h^{\tau }})}\big \}_{m-1}\big )\in P_{p_K}(K)$ is the $L^2(K)$-projection weighted by $\chi _K(x)$ given by

55 $$\begin{aligned} \big ({P_h\big (\big \{{{\vartheta }({u_h^{\tau }})}\big \}_{m-1}\big )},{\chi _K v}\big )_{K} =\big ({ \big \{{{\vartheta }({u_h^{\tau }})}\big \}_{m-1}},{\chi _K v}\big )_{K} \qquad \forall v \in P_{p_K}(K). \end{aligned}$$

We note that

56 $$\begin{aligned} {P_h\big (\big \{{{\vartheta }({u_h^{\tau }})}\big \}_{m-1}\big )} \not = \big \{{\overline{{\vartheta }({u_h^{\tau }})}}\big \}_{m-1}, \end{aligned}$$

in general, compare with (52).

Using the inverse inequality, the polynomial function *w* given by (54) can be estimated as

57 $$\begin{aligned} {\left\| w\right\| }_{V_{K,m}}^{2}&= {{d_{K,m}^{-2}}} \left( h_K^2{\left\| \nabla w\right\| }_{K,m}^{2} +\tau _m^2 {\left\| w^{\prime }\right\| }_{K,m}^{2} \right) \lesssim {{d_{K,m}^{-2}}} {\left\| w\right\| }_{K,m}^{2} \\&\le \frac{{d_{K,m}^{2}}}{\tau _m^2} {\left\| P_h\big (\big \{{{\vartheta }({u_h^{\tau }})}\big \}_{m-1}\big )\right\| }_{K}^{2} \int _{I_m} \varPhi _m^2(t) {\,{\mathrm d}t}\lesssim \frac{{d_{K,m}^{2}}}{\tau _m} {\left\| P_h\big (\big \{{{\vartheta }({u_h^{\tau }})}\big \}_{m-1}\big )\right\| }_{K}^{2} . \nonumber \end{aligned}$$

Similarly as in [11] or [18], we introduce the oscillation terms

58 $$\begin{aligned} \eta _{G,K,m}&:= {d_{K,m}}{\left\| {\overline{g}} - g\right\| }_{K,m} , \quad \eta _{{\vartheta },K,m}:= \frac{{d_{K,m}}}{\sqrt{\tau _m}}{\left\| \big \{{{\vartheta }({u_h^{\tau }})}\big \}_{m-1} -P_h\big (\big \{{{\vartheta }({u_h^{\tau }})}\big \}_{m-1}\big )\right\| }_{K} ,\\ \eta _{{\vartheta }^\prime ,K,m}&:= {d_{K,m}}{\left\| \overline{\vartheta ^\prime ({u_h^{\tau }})}- {\vartheta }^\prime ({u_h^{\tau }}) \right\| }_{K,m} , \nonumber \\ \eta _{\sigma ,K,m}&:=\frac{{d_{K,m}}}{{h_K}} {\left\| {\overline{\sigma }}- {\sigma }({u_h^{\tau }}, \nabla {u_h^{\tau }})\right\| }_{K,m} +{d_{K,m}}{\left\| \nabla \cdot {\overline{\sigma }}- \nabla \cdot {\sigma }({u_h^{\tau }}, \nabla {u_h^{\tau }})\right\| }_{K,m} .\nonumber \end{aligned}$$

The goal is to prove the lower bounds of the proposed error estimates, namely to estimate $\eta _{T,K,m}$, $\eta _{R,K,m}$ and $\eta _{S,K,m}$ by ${{\mathcal {R}}}_{K,m}({u_h^{\tau }})$ and the oscillation terms (58), $K\in {{\mathcal {T}}_h}$, $m=1,\dots ,r$.

#### Theorem 2

Let $\eta _{T,K,m}$, $K\in {{\mathcal {T}}_h^m}$, $m=1,\dots , r$ be the error estimates given by (42), then

59 $$\begin{aligned} \eta _{T,K,m}\lesssim {{\mathcal {R}}}_{K,m}({u_h^{\tau }}) + \eta _{G,K,m}+ \eta _{{\vartheta }^\prime ,K,m}+\eta _{{\vartheta },K,m}+ \eta _{S,K,m}. \end{aligned}$$

where ${{\mathcal {R}}}_{K,m}$ are the local error measures defined by (49)–(50) and the oscillation terms $\eta _{G,K,m}$, $\eta _{{\vartheta },K,m}$ and $\eta _{{\vartheta }^\prime ,K,m}$ are given by (58).

#### Proof

We start the proof by the putting function *w* from (54) as the test function in (50), i.e.

60 $$\begin{aligned} {{\mathcal {R}}}_{K,m}({u_h^{\tau }})=\sup _{0\ne v\in V_{K,m}}\frac{\sum _{K,m}{b_{K,m}}({u_h^{\tau }},v)}{{\left\| v\right\| }_{{V^\tau }} } \ge \frac{{b_{K,m}}({u_h^{\tau }},w)}{{\left\| w\right\| }_{{V^\tau }} } \end{aligned}$$

since $\text{ supp } (w) = K\times I_m$, cf. (54). Then, using (20) and the fact that *w* vanishes on ${\partial K}$, we have

61 $$\begin{aligned} {{\mathcal {R}}}_{K,m}({u_h^{\tau }})&\ge \frac{ \big ({{\vartheta }^\prime ({u_h^{\tau }})-g},{w}\big )_{K,m} + \big ({{\sigma }({u_h^{\tau }}, \nabla {u_h^{\tau }})},{\nabla w}\big )_{K,m} + \big ({ \big \{{{\vartheta }({u_h^{\tau }})}\big \}_{m-1}},{w^{m-1}_{+}}\big )_{K} }{ {\left\| w \right\| }_{V_{K,m}} } \\&= \frac{ \big ({\overline{\vartheta ^\prime ({u_h^{\tau }})}- {\overline{g}}},{w}\big )_{K,m} + \big ({{\sigma _h^{\tau }}},{\nabla w}\big )_{K,m} }{ {\left\| w \right\| }_{V_{K,m}} } + \frac{\big ({ \big \{{{\vartheta }({u_h^{\tau }})}\big \}_{m-1}},{ w^{m-1}_{+}}\big )_{K} }{ {\left\| w \right\| }_{V_{K,m}} } =:\xi _1 + \xi _2 \nonumber \\&\quad + \frac{ \big ({{\overline{g}}- g},{w}\big )_{K,m} + \big ({{\sigma }- {\sigma _h^{\tau }}},{\nabla w}\big )_{K,m} + \big ({ {\vartheta }^\prime ({u_h^{\tau }}) - \overline{\vartheta ^\prime ({u_h^{\tau }})}},{w}\big )_{K,m} }{ {\left\| w \right\| }_{V_{K,m}} }\nonumber \\&=:\xi _3 + \xi _4 + \xi _5. \nonumber \end{aligned}$$

The functions $\overline{\vartheta ^\prime ({u_h^{\tau }})}$, ${\overline{g}}$ and ${\sigma _h^{\tau }}$ are polynomials of degree *q* in time whereas *w* and $\nabla w$ are the (Legendre) polynomial of degree $(q+1)$ in time, cf. (54). Due to the $L^2(I_m)$-orthogonality of the Legendre polynomials, we have $\xi _1=0$, since

62 $$\begin{aligned} \big ({\overline{\vartheta ^\prime ({u_h^{\tau }})}- {\overline{g}}},{w}\big )_{K,m} + \big ({{\sigma _h^{\tau }}},{\nabla w}\big )_{K,m} = 0 \end{aligned}$$

Moreover, using inequality (57), relations (54)-(55) and the equivalence of norms on finite dimensional spaces,

we obtain

63 $$\begin{aligned} \xi _2& > rsim \frac{ \big ({P_h\big (\big \{{{\vartheta }({u_h^{\tau }})}\big \}_{m-1}\big )},{ \frac{{d_{K,m}^{2}}}{\tau _m} P_h\big (\big \{{{\vartheta }({u_h^{\tau }})}\big \}_{m-1}\big )\chi _K}\big )_{K} }{ \frac{{d_{K,m}}}{\sqrt{\tau _m}} {\left\| P_h\big (\big \{{{\vartheta }({u_h^{\tau }})}\big \}_{m-1}\big )\right\| }_{K} }\\& > rsim \frac{{d_{K,m}}}{\sqrt{\tau _m}} \frac{ \big ({P_h\big (\big \{{{\vartheta }({u_h^{\tau }})}\big \}_{m-1}\big )},{P_h\big (\big \{{{\vartheta }({u_h^{\tau }})}\big \}_{m-1}\big )}\big )_{K}}{{\left\| P_h\big (\big \{{{\vartheta }({u_h^{\tau }})}\big \}_{m-1}\big )\right\| }_{K} } = \frac{{d_{K,m}}}{\sqrt{\tau _m}} {{\left\| P_h\big (\big \{{{\vartheta }({u_h^{\tau }})}\big \}_{m-1}\big )\right\| }_{K} }. \nonumber \end{aligned}$$

Furthermore, let $w_{K,m} = \frac{1}{K\times I_m} \int _{K\times I_m} w {\,{\mathrm d}x}{\,{\mathrm d}t}$ be the mean value of *w* on the space-time element $K\times I_m$. Due to (52), the Cauchy–Schwarz inequality and (35), we have

64 $$\begin{aligned} |\xi _3|&=\frac{\big | \big ({{\overline{g}}- g},{w-w_{K,m}}\big )_{K,m} \big |}{ {\left\| w \right\| }_{V_{K,m}} }\nonumber \\&\le \frac{ {\left\| {\overline{g}}- g\right\| }_{K,m} {\left\| w-w_{K,m}\right\| }_{K,m} }{ {\left\| w \right\| }_{V_{K,m}} } \lesssim {d_{K,m}}{\left\| {\overline{g}}- g\right\| }_{K,m} = \eta _{G,K,m}, \end{aligned}$$

and

65 $$\begin{aligned}&|\xi _5| \lesssim {d_{K,m}}{\left\| {\vartheta }^\prime ({u_h^{\tau }}) - \overline{\vartheta ^\prime ({u_h^{\tau }})}\right\| }_{K,m} = \eta _{{\vartheta }^\prime ,K,m}. \end{aligned}$$

Similarly, the Cauchy–Schwarz inequality and (22) imply

66 $$\begin{aligned} |\xi _4|&\le \frac{{d_{K,m}}}{{h_K}} {\left\| {\sigma }({u_h^{\tau }}, \nabla {u_h^{\tau }}) - {\sigma _h^{\tau }}\right\| }_{K,m} \, \frac{ {h_K}{\left\| \nabla w\right\| }_{K,m} }{ {d_{K,m}}{\left\| w \right\| }_{V_{K,m}} } \nonumber \\&\le \frac{{d_{K,m}}}{{h_K}} {\left\| {\sigma }({u_h^{\tau }}, \nabla {u_h^{\tau }}) - {\sigma _h^{\tau }}\right\| }_{K,m} = \eta _{S,K,m}. \end{aligned}$$

Collecting (61)–(66), we have

67 $$\begin{aligned} {{\mathcal {R}}}_{K,m}({u_h^{\tau }})& > rsim \frac{{d_{K,m}}}{\sqrt{\tau _m}} {{\left\| P_h\big (\big \{{{\vartheta }({u_h^{\tau }})}\big \}_{m-1}\big )\right\| }_{K} } - \eta _{G,K,m}-\eta _{S,K,m}-\eta _{{\vartheta }^\prime ,K,m}. \end{aligned}$$

Moreover, using (42c), (26), integration by parts, the boundedness of the Radau polynomials, the triangle inequality and (58), we have

68 $$\begin{aligned} \eta _{T,K,m}&= \frac{{d_{K,m}}}{\tau _m} {\left\| {R_h^{\tau }}- {\vartheta }({u_h^{\tau }})\right\| }_{K,m} = \frac{{d_{K,m}}}{\tau _m} {\left\| \big \{{{\vartheta }({u_h^{\tau }})}\big \}_{m-1} r_m\right\| }_{K,m} \\&= \frac{{d_{K,m}}}{\tau _m} {\left\| \big \{{{\vartheta }({u_h^{\tau }})}\big \}_{m-1}\right\| }_{K} \sqrt{ \int _{I_m} r_m^2 {\,{\mathrm d}t}}\lesssim \frac{{d_{K,m}}}{\sqrt{\tau _m}}{\left\| \big \{{{\vartheta }({u_h^{\tau }})}\big \}_{m-1}\right\| }_{K} \nonumber \\&\le \frac{{d_{K,m}}}{\sqrt{\tau _m}}{\left\| P_h\big (\big \{{{\vartheta }({u_h^{\tau }})}\big \}_{m-1}\big )\right\| }_{K} + \eta _{{\vartheta },K,m}. \nonumber \end{aligned}$$

Hence, (67) and (68)

69 $$\begin{aligned} \eta _{T,K,m}\le {{\mathcal {R}}}_{K,m}({u_h^{\tau }}) + \eta _{{\vartheta },K,m}+ \eta _{G,K,m}+ \eta _{{\vartheta }^\prime ,K,m}+ \eta _{S,K,m}, \end{aligned}$$

which proves the theorem. $\square$

#### Theorem 3

Let $\eta _{S,K,m}$ and $\eta _{R,K,m}$, $K\in {{\mathcal {T}}_h^m}$, $m=1,\dots , r$ be the error estimates given by (42), then

70 $$\begin{aligned} \eta _{R,K,m}&\lesssim {{\mathcal {R}}}_{{\omega _K},m}({u_h^{\tau }}) + \eta _{G,K,m}+ \eta _{\sigma ,K,m}+ \eta _{S,K,m}, \end{aligned}$$

71 $$\begin{aligned} \eta _{S,K,m}&\lesssim {{\mathcal {R}}}_{{\omega _K},m}({u_h^{\tau }}) + \eta _{G,K,m}+ \sum _{K\subset {\omega _K}}\eta _{\sigma ,K,m}, \end{aligned}$$

where ${{\mathcal {R}}}_{{\omega _K},m}$ is the local error measures defined by (49)–(50) and the oscillation terms $\eta _{G,K,m}$, $\eta _{{\vartheta },K,m}$ and $\eta _{{\vartheta }^\prime ,K,m}$ are given by (58).

#### Proof

The proof is in principle identical with the proof [18, Lemmas 7-9], we present the main step for completeness. Let ${\overline{g}}$ and ${\overline{\sigma }}$ be the projection given by (52) and (53). Using the triangle inequality, the inverse inequality and (58), we obtain

72 $$\begin{aligned} \eta _{R,K,m}&= {d_{K,m}}{\left\| ({R_h^{\tau }})^\prime - \nabla \cdot {\sigma _h^{\tau }}- g \right\| }_{K,m} \\&\le {d_{K,m}}{\left\| ({R_h^{\tau }})^\prime - \nabla \cdot {\overline{\sigma }}- {\overline{g}}\right\| }_{K,m} + {d_{K,m}}{\left\| {\overline{g}}-g\right\| }_{K,m} + {d_{K,m}}{\left\| \nabla \cdot {\overline{\sigma }}-\nabla \cdot {\sigma _h^{\tau }}\right\| }_{K,m} \nonumber \\&\lesssim {d_{K,m}}{\left\| ({R_h^{\tau }})^\prime - \nabla \cdot {\overline{\sigma }}- {\overline{g}}\right\| }_{K,m} + \eta _{G,K,m}+ \frac{{d_{K,m}}}{{h_K}} {\left\| {\overline{\sigma }}- {\sigma _h^{\tau }}\right\| }_{K,m} . \nonumber \end{aligned}$$

The first term on the right-hand side of (72) can be estimated as in [36, Theorem 4.10] by

73 $$\begin{aligned} {d_{K,m}}{\left\| ({R_h^{\tau }})^\prime - \nabla \cdot {\overline{\sigma }}- {\overline{g}}\right\| }_{K,m} \lesssim Res_{{\omega _K}, m}({u_h^{\tau }})+\eta _{G,K,m}+\eta _{\sigma ,K,m}, \end{aligned}$$

where the resulting oscillation terms are estimated with the aid (58). Moreover, the last term on the right-hand side of (72) together with (42b) and assumption (58), reads

74 $$\begin{aligned} \frac{{d_{K,m}}}{{h_K}} {\left\| {\overline{\sigma }}- {\sigma _h^{\tau }}\right\| }_{K,m}&\le \frac{{d_{K,m}}}{{h_K}} {\left\| {\overline{\sigma }}- {\sigma }({u_h^{\tau }},\nabla {u_h^{\tau }}) \right\| }_{K,m} + \frac{{d_{K,m}}}{{h_K}} {\left\| {\sigma }({u_h^{\tau }},\nabla {u_h^{\tau }}) - {\sigma _h^{\tau }}\right\| }_{K,m} \\&\le \eta _{\sigma ,K,m}+ \eta _{S,K,m}, \nonumber \end{aligned}$$

which proves (70).

The proof of (71) is based on the decomposition

75 $$\begin{aligned} {\left\| {\sigma _h^{\tau }}- {\sigma }({u_h^{\tau }}, \nabla {u_h^{\tau }})\right\| }_{K,m} \le {\left\| {\sigma _h^{\tau }}- {\overline{\sigma }}\right\| }_{K,m} + {\left\| {\overline{\sigma }}- {\sigma }({u_h^{\tau }}, \nabla {u_h^{\tau }})\right\| }_{K,m} . \end{aligned}$$

While the second term on the right-hand side of (75) can be estimated by assumption (58), the estimate of the first term is somewhat more technical. It depends on the flux reconstruction used. For the flux reconstruction in Sect. 5.2, the proof is identical to the proof of [18, Lemma 9], which mimics the stationary variant [24, Theorem 3.12]. On the other hand, using the flux reconstruction from Sect. 5.1, it is possible to apply the technique from [11, Lemma 7.5], where the final relation has to be integrated over $I_m$. $\square$

## Spatial Flux Reconstructions and Stopping Criteria

We present two ways of reconstructing the spatial flux ${\sigma _h^{\tau }}\in L^2(0,T,H(\textrm{div},\varOmega ))$ that satisfies the assumptions (33)–(34). The first one, proposed in [19] for the case of homogeneous Dirichlet boundary condition, is defined by the volume and edge momenta of the Raviart-Thomas-Nedelec (RTN) elements, cf. [7], and is easy to compute. The second approach is based on the solution of local Neumann problems on patches associated with each vertex of the mesh. This idea comes from, e.g., [24], its space-time variant was proposed in [18] for nonlinear convection-diffusion equations. Finally, in Sect. 5.3, we discuss the errors arising from the solution of algebraic systems and introduce a stopping criterion for the appropriate iterative solver.

### Element-Wise Variant

We denote by $p_{K,\max }$ the maximum polynomial degree over the element *K* and its neighbours that share the entire edge with *K* and $p_{\gamma ,\max }$ the maximum polynomial degree on neighbouring elements having a common edge $\gamma$. Let ${\textrm{RTN}}_{p_{K,\max }}(K)$ be the space of RTN finite elements of order $p_{K,\max }$ for element $K\in {{\mathcal {T}}_h^m}$, cf. (32), and ${u_h^{\tau }}\in {{S_{hp}^{\tau q}}}$ be the approximate solution. The spatial reconstruction ${\sigma _h^{\tau }}$ is defined element-wise: for each $K\in {{\mathcal {T}}_h^m}$, find ${{\sigma _h^{\tau }}}|_{K\times I_m}\in P_q(I_m,{\textrm{RTN}}_{p_{K,\max }}(K))$ with ${{\sigma _h^{\tau }}\cdot n}|_{\gamma \times I_m}\in P_q(I_m,P_{p_{\gamma ,\max }}(\gamma ))$ such that

76 $$\begin{aligned} \big ({{\sigma _h^{\tau }}\cdot n},{v}\big )_{\gamma ,m}&= {\left\{ \begin{array}{ll} \big ({\left\langle {{\textbf{K}}({u_h^{\tau }}) \nabla {u_h^{\tau }}}\right\rangle \cdot {n} - \alpha [{{u_h^{\tau }}}]\cdot {n}},{v}\big )_{\gamma ,m} & \quad \forall v\in P_q(I_m,P_{p_{\gamma ,\max }}(\gamma )), \ \gamma \subset {{\partial K}\setminus {\varGamma _\textrm{N}}}\\ \big ({g_N},{v}\big )_{\gamma } & \quad \forall v\in P_q(I_m,P_{p_{\gamma ,\max }}(\gamma )), \ \gamma \subset {{\partial K}_{\!N}}\\ \end{array}\right. } \\ \big ({{\sigma _h^{\tau }}},{v}\big )_{K,m}&=\big ({{\textbf{K}}({u_h^{\tau }})\nabla {u_h^{\tau }}},{\nabla v}\big )_{K,m} +(\beta -\tfrac{1}{2}) \big ({ {\textbf{K}}({u_h^{\tau }})[{{u_h^{\tau }}}]},{ \nabla v}\big )_{{{\partial K}\setminus {\varGamma }},m} \nonumber \\&\quad +(2\beta -1) \big ({ {\textbf{K}}({u_h^{\tau }})[{{u_h^{\tau }}}]},{ \nabla v}\big )_{{{\partial K}\cap {\varGamma _\textrm{D}}},m} \qquad \forall v\in P_q(I_m,P_{p_{K,\max }-1}(K)^d). \nonumber \end{aligned}$$

The edge momenta in (76) are uniquely defined and since $p_{\gamma ,\max }\le p_{K,\max }$, ${\sigma _h^{\tau }}$ in (76) is well defined as well. Here, the numerical flux $\left\langle {{\textbf{K}}({u_h^{\tau }}) \nabla {u_h^{\tau }}}\right\rangle \cdot {n} - \alpha [{{u_h^{\tau }}}]\cdot {n}$ is conservative on interior edges, which implies that ${\sigma _h^{\tau }}\cdot {n}$ are the same on each interior edge $\gamma$ and therefore the resulting reconstruction ${{\sigma _h^{\tau }}}\in L^2(0,T,H(\textrm{div},\varOmega ))$ globally.

Obviously, the first relation in (76) with $p_K\le p_{\gamma ,\max }$ directly implies assumption (34). Moreover, using the Green theorem, (76), (12), (31) and $p_K\le p_{\gamma ,\max }\le p_{K,\max }$, we obtain

77 $$\begin{aligned} \big ({\nabla \cdot {{\sigma _h^{\tau }}}},{v}\big )_{K,m}&= - \big ({{\sigma _h^{\tau }}},{\nabla v}\big )_{K,m} + \big ({{\sigma _h^{\tau }}\cdot {n_K}},{v}\big )_{{\partial K},m}\\&= - \big ({{\textbf{K}}({u_h^{\tau }})\nabla {u_h^{\tau }}},{\nabla v}\big )_{K,m} + \big ({\left\langle {{\textbf{K}}({u_h^{\tau }}) \nabla {u_h^{\tau }}}\right\rangle \cdot {n} - \alpha [{{u_h^{\tau }}}]\cdot {n}},{v}\big )_{{{\partial K}\setminus {\varGamma _\textrm{N}}},m} \nonumber \\&\quad - (\beta -\tfrac{1}{2}) \big ({{\textbf{K}}( {u_h^{\tau }})[{ {u_h^{\tau }}}]},{ \nabla v}\big )_{{{\partial K}\setminus {\varGamma }},m} - (2\beta -1) \big ({{\textbf{K}}( {u_h^{\tau }})[{ {u_h^{\tau }}}]},{ \nabla v}\big )_{{{\partial K}\cap {\varGamma _\textrm{D}}},m} \nonumber \\&\quad +\big ({g_N},{v}\big )_{{{\partial K}_{\!N}},m} \nonumber \\&=-{A_{K,m}}({u_h^{\tau }},v) - \big ({g},{v}\big )_{K,m} \!=\! \big ({({R_h^{\tau }})^{\prime } - g },{v}\big )_{K,m}\nonumber \\&\ \forall v\in P_q(I_m,P_{p_K}(K)),\, K\!\in \!{{\mathcal {T}}_h^m}\nonumber , \end{aligned}$$

which justifies the assumption (33).

### Patch-Wise Variant

For each vertex *a* of the mesh ${{\mathcal {T}}_h^m}$, we denote by ${\omega _a}$ a patch of elements $K\in {{\mathcal {T}}_h^m}$ sharing this vertex. By $p_a= \max _{K\in {\omega _a}} p_K$ we denote the maximal polynomial degree on ${\omega _a}$. Let $P_{p_a}^{*}({\omega _a})$ be the space of piecewise polynomial discontinuous functions of degree $p_a$ on ${\omega _a}$ with mean value zero for $a \notin \partial {\varOmega }$. We define the space

78 $$ \begin{aligned} W^N_{{\textrm{RTN}},p_a}({\omega _a})&= \{ v \in H(\textrm{div},{\omega _a}); v|_K \in {\textrm{RTN}}_{p_a}(K), v \cdot n = 0 \text{ on } \partial {\omega _a}\}, \quad a \notin \partial {\varOmega }\\ W^N_{{\textrm{RTN}},p_a}({\omega _a})&= \{ v \in H(\textrm{div},{\omega _a}); v|_K \in {\textrm{RTN}}_{p_a}(K), v \cdot n = 0 \text{ on } \partial {\omega _a}\setminus \partial {\varOmega }, \nonumber \\&\quad  \& \ (v \cdot n,\phi )_{\gamma ,m} = (g_N,\phi )_{\gamma ,m}\ \forall \phi \in P_q(I_m, P_{p_a}(\gamma )) \text{ on } \partial {\omega _a}\cap {{\partial K}_{\!N}}\},a \in \partial {\varOmega }. \nonumber \end{aligned}$$

We set the local problems on patches ${\omega _a}$ for all vertices *a*: find ${\sigma _h^{\tau }}\in P_q(I_m, W^N_{{\textrm{RTN}},p_a}({\omega _a}))$ and $r^\tau _a \in P_q(I_m, P_{p_a}^{*}({\omega _a}))$ such that

79 $$\begin{aligned} \big ({{\sigma }^\tau _a},{v}\big )_{{\omega _a}, m} - \big ({r^\tau _a},{\nabla \cdot v}\big )_{{\omega _a}, m}&=\big ({\xi ^1_a},{v}\big )_{{\omega _a}, m} \quad \forall v \in P_q(I_m, W^N_{{\textrm{RTN}},p_a}({\omega _a})) \\ \big ({\nabla \cdot {\sigma }^\tau _a},{\phi }\big )_{{\omega _a}, m}&= \big ({\xi ^2_a},{\phi }\big )_{{\omega _a}, m} \quad \forall \phi \in P_q(I_m, P_{p_a}^{*}({\omega _a})),\nonumber \end{aligned}$$

where

80 $$\begin{aligned} \xi ^1_a&= \psi _a {\sigma }({u_h^{\tau }}, \nabla {u_h^{\tau }}) \\ \xi ^2_a&= \psi _a ({R_h^{\tau }})^\prime - \psi _a g + \nabla \psi _a \cdot \mathbf {\xi }({u_h^{\tau }}, \nabla {u_h^{\tau }}), \nonumber \end{aligned}$$

with

81 $$\begin{aligned} \mathbf {\xi }({u_h^{\tau }}, \nabla {u_h^{\tau }}) = {\sigma }({u_h^{\tau }}, \nabla {u_h^{\tau }}) + (2\beta -1)\sum _{\gamma \not \subset {\varGamma _\textrm{N}}}\ell _{m,\gamma }({u_h^{\tau }}), \end{aligned}$$

and $\ell _{m,\gamma }: {S_{hp,m}}\rightarrow [{S_{h0,m}}]^d$ is the lifting operator defined by

82 $$\begin{aligned}&\int _{\varOmega } \ell _{m,\gamma }({u_h^{\tau }}) \cdot v{\,{\mathrm d}x}= \int _{\gamma } [{{u_h^{\tau }}}] \left\langle {{\textbf{K}}({u_h^{\tau }})v}\right\rangle {\,{\mathrm d}x}\qquad \forall v \in [{S_{h0,m}}]^d, \quad \gamma \not \subset {\varGamma _\textrm{N}}. \end{aligned}$$

Then the final reconstructed flux is obtained by summing up ${\sigma }^\tau _a$ on each element that contains vertex *a*, i.e.,

83 $$\begin{aligned} {\sigma _h^{\tau }}|_{K,m} = \sum _{a \in K} {\sigma }^\tau _a |_K . \end{aligned}$$

The assumption (34) follows directly from (78) and $p_K\le p_a$. Inserting the hat function $\psi _a v$ for $a\not \in \partial \varOmega$ and $v\in P_q(I_m)$ in (17), using (5), (82) and omitting the zero terms, we have

84 $$\begin{aligned}&\sum _{K\in {{\mathcal {T}}_h^m}} {A_{K,m}}({u_h^{\tau }},\psi _av)\nonumber \\&\quad =\sum _{K\in {{\mathcal {T}}_h^m}}\big ({{\textbf{K}}({u_h^{\tau }})\nabla {u_h^{\tau }}},{\nabla \psi _a v}\big )_{K,m}\\&\qquad +(2\beta -1)\sum _{\gamma \not \subset \partial \varOmega }\big ({[{{u_h^{\tau }}}] },{ \left\langle {{\textbf{K}}({u_h^{\tau }})\nabla \psi _a v}\right\rangle }\big )_{\gamma ,m}+ (2\beta -1)\sum _{\gamma \subset {\varGamma _\textrm{D}}}\big ({[{{u_h^{\tau }}}] },{ {\textbf{K}}({u_h^{\tau }})\nabla \psi _a v}\big )_{\gamma ,m} \nonumber \\&\qquad -\big ({g},{\psi _a v}\big )_{\varOmega ,m} =\big ({\xi ^2_a},{v}\big )_{{\omega _a}, m} - \big ({{R_h^{\tau }}},{\psi _a v}\big )_{{\omega _a}, m} \nonumber \end{aligned}$$

Applying (13) and (31), we gain for $a\not \in \partial \varOmega$ and $v\in P_q(I_m)$

85 $$\begin{aligned} \big ({\nabla \cdot {\sigma }^\tau _a},{v}\big )_{{\omega _a}, m}= \sum _{K\subset {\omega _a}}\left( {A_{K,m}}({u_h^{\tau }},\psi _av)+ \big ({{R_h^{\tau }}},{\psi _a v}\big )_{K, m}\right) =\big ({\xi ^2_a},{v}\big )_{{\omega _a}, m}. \end{aligned}$$

From this it follows that the second relation in (79) holds element-wise, i.e.

86 $$\begin{aligned} \big ({\nabla \cdot {\sigma }^\tau _a},{\phi }\big )_{K, m} = \big ({\xi ^2_a},{\phi }\big )_{K, m},\quad \forall \phi \in P_q(I_m, P_{p_a}(K)). \end{aligned}$$

Then (33) follows from

87 $$\begin{aligned} (\nabla \cdot {\sigma _h^{\tau }}, \phi )_{K,m}&= \sum _{a\subset K}(\nabla \cdot {\sigma }^\tau _a, \phi )_{K,m}= \sum _{a\subset K}\big ({\xi ^2_a},{\phi }\big )_{K, m}\\&= (({R_h^{\tau }})^\prime - g, \phi )_{K,m} \quad \forall \phi \in P_q(I_m, P_{p_a}(K)) \nonumber \end{aligned}$$

and from $$p_K\le p_a$$.

### Stopping Criteria for Iterative Solvers

The space-time discretization (13) leads to a system of nonlinear algebraic equations for each time level $m=1,\dots ,r$. These systems have to be solved iteratively by a suitable solver, e.g., the Picard method, the Newton method or their variants. Therefore, it is necessary to set a suitable stopping criterion for the iterative solvers so that, on the one hand, the algebraic errors do not affect the quality of the approximate solution and, on the other hand, an excessive number of algebraic iterations is avoided.

However, the error estimates presented in Sect. 4 do not take into account errors arising from the inaccurate solution of these systems. Indeed, the aforementioned reconstructions fulfill assumption (33) only if the systems given by (13) are solved exactly. The full a posteriori error analysis including algebraic errors has been treated, e.g., in [8, 23, 29]. These error estimators are based on additional flux reconstructions that need to be evaluated at each iteration, and therefore, the overall computational time is increased.

To speed up the computations and control the algebraic errors, we adopt the technique of [17]. This approach offers (i) the measurement of algebraic errors by a quantity similar to the error measure (23), (ii) the setting of the stopping criterion for iterative solvers with one parameter corresponding to the relative error, and (iii) a fast evaluation of the required quantities.

For each $m=1,\dots ,r$, we define the estimators (cf. (23))

88 $$\begin{aligned} {\eta _{\textrm{alg}}^m}({u_h^{\tau }}) = \sup _{0\ne v\in {S_{hp}^{\tau q}}}\frac{\sum _{K\in {{\mathcal {T}}_h^m}}{b_{K,m}}({u_h^{\tau }},v)}{{\left\| v\right\| }_{{V^\tau }} },\qquad {\eta _{\textrm{spa}}^m}({u_h^{\tau }}) = \sup _{0\ne v\in {S_{hp+1}^{\tau q+1}}}\frac{\sum _{K\in {{\mathcal {T}}_h^m}}{b_{K,m}}({u_h^{\tau }},v)}{{\left\| v\right\| }_{{V^\tau }} }, \end{aligned}$$

where the norm ${\left\| \cdot \right\| }_{{V^\tau }}$ is given by (22),

89 $$\begin{aligned}&{S_{hp+1}^{\tau q+1}}=\{v\in L^2({\varOmega }\times (0,T)):\ v|_{I_m}\in P_{q+1}(I_m,{S_{hp+1,m}}),\ m=1,\dots , r\},\\&\quad \text{ and }\quad {S_{hp+1,m}}=\{v\in L^2({\varOmega }):v|_K\in P_{p_K+1}(K),\ K\in {{\mathcal {T}}_h^m}\},\qquad m=0,\dots ,r.\nonumber \end{aligned}$$

The space ${S_{hp+1}^{\tau q+1}}$ is an enrichment space of ${S_{hp}^{\tau q}}$ by polynomials of the space degree $p_K+1$ and the time degree $q+1$ for each $K\times I_m$, $K\in {{\mathcal {T}}_h^m}$, $m=1,\dots ,r$. Finally, we define the global in time quantities

90 $$\begin{aligned} {\eta _{\textrm{alg}}}({u_h^{\tau }}) = \left( \sum _{m=1}^r ({\eta _{\textrm{alg}}^m}({u_h^{\tau }}))^2\right) ^{1/2}, \qquad {\eta _{\textrm{spa}}}({u_h^{\tau }}) = \left( \sum _{m=1}^r ({\eta _{\textrm{spa}}^m}({u_h^{\tau }}))^2\right) ^{1/2}. \end{aligned}$$

Obviously, if ${u_h^{\tau }}$ fulfills (13) exactly, then ${\eta _{\textrm{alg}}^m}({u_h^{\tau }})=0$ for all $m=0,\dots ,r$. Moreover, if ${u_h^{\tau }}$ is the weak solution (6) then ${\eta _{\textrm{spa}}^m}({u_h^{\tau }})=0$ for all $m=0,\dots ,r$. Comparing (88) with (23), the quantity ${\eta _{\textrm{spa}}}({u_h^{\tau }})$ exhibits a variant of the error measure ${{\mathcal {R}}}({u_h^{\tau }})$. Nevertheless, ${\eta _{\textrm{spa}}}({u_h^{\tau }})$ is neither lower nor upper bound of ${{\mathcal {R}}}({u_h^{\tau }})$ since ${S_{hp+1}^{\tau q+1}}\not \subset {V^\tau }$ and ${V^\tau }\not \subset {S_{hp+1}^{\tau q+1}}$.

The quantities (88) can be evaluated very fast since the suprema (maxima) are the sum of the suprema (maxima) for all space-time elements $K\times I_m$, $K\in {{\mathcal {T}}_h^m}$, $m=1,\dots ,r$, which are computable separately, cf. [17] for details. Hence, we prescribe the stopping criterion for the corresponding iterative solver as

91 $$\begin{aligned} {\eta _{\textrm{alg}}^m}({u_h^{\tau }}) \le c_A{\eta _{\textrm{spa}}^m}({u_h^{\tau }}),\qquad m=1,\dots ,r, \end{aligned}$$

where $c_A\in (0,1)$ is the user-dependent constant. The justification of this approach and the influence of algebraic errors on the error estimates are studied numerically in Sect. 6.1.1.

## Numerical Experiments

We present numerical experiments that justify the a posteriori error estimates (41)–(42). Since the error measure (23) is the dual norm of the residual, it is not possible to evaluate the error even if the exact solution is known. Therefore, similarly to [18], we approximate the error by solving the dual problem given for each time interval $I_m,\ m=1,\dots ,r$: Find $\psi _m \in {Y^{\tau }_{m}}=L^2(I_m,H^1({\varOmega }))$,

92 $$\begin{aligned} \big ({\psi _m},{\phi }\big )_{{Y^{\tau }_{m}}} = \sum _{K,m} {b_{K,m}}(u^{\tau }_h, \phi ) \qquad \forall \phi \in {Y^{\tau }_{m}}, \end{aligned}$$

where (cf. (21a)–(22))

93 $$\begin{aligned} \big ({u},{v}\big )_{{Y^{\tau }_{m}}}= \sum \nolimits _{K\in {{\mathcal {T}}_h^m}}{{d_{K,m}^{-2}}}\left( h_K^2\big ({\nabla u},{\nabla v}\big )_{K,m} +\tau _m^2 \big ({u^\prime },{v^{\prime }}\big )_{K,m}\right) ,\qquad m=1,\dots ,r. \end{aligned}$$

Then we have ${{\mathcal {R}}}({u_h^{\tau }})^2 = \sum ^r_{m=1}{\left\| \psi \right\| }_{{Y^{\tau }_{m}}}^{2}$. We solve (92) for each $m=1,\dots ,r$ by linear conforming finite element on a global refinement of the space-time mesh ${{\mathcal {T}}_h^m}\times I_m$ which is proportional to the space and time polynomial approximation degrees. We denote this quantity by $\widetilde{{{\mathcal {R}}}}({u_h^{\tau }})$. The second error contribution ${{\mathcal {J}}}$ given by (24) is computable, so the total error ${{\mathcal {E}}}$ (cf. (25)) is approximated by $\widetilde{{{\mathcal {E}}}}({u_h^{\tau }}):= \left( \widetilde{{{\mathcal {R}}}}({u_h^{\tau }})^2+{{\mathcal {J}}}({u_h^{\tau }})\right) ^{1/2}$.

### Remark 3

Sometimes, this approximate evaluation of the (exact) error is not sufficiently accurate for fine grids and high polynomial approximation degrees. In this case, very fine global refinement is required and then the resulting algebraic systems are too large to be solved in a reasonable time.

All numerical experiments were carried out using the patch-wise reconstruction from Sect. 5.2 using the in-house code ADGFEM [10]. The arising nonlinear algebraic systems are solved iteratively by a Newton-like method, we refer to [14] for details. Each Newton-line iteration leads to a linear algebraic system that is solved by GMRES method with block ILU(0) preconditioner.

### Barenblatt Problems

We consider two nonlinear variants of (3) following from the Barenblatt problem [4] where the analytical solution exists. The first variant reads

94 $$\begin{aligned} \partial _t{{\vartheta }(u)} - \varDelta u = 0, \qquad {\vartheta }(u) = u^{1/m}, \qquad m\in (0,1), \end{aligned}$$

where the analytical solution is

95 $$\begin{aligned} u(x_1,x_2,t) = \frac{1}{1+t} \bigg (\bigg \lfloor [1 - \frac{m-1}{4m^2} \frac{x_1^2+x_2^2}{(1+t)^{1/m}} \bigg \rfloor _{+}\bigg )^{ \frac{m}{m-1} }, \qquad \lfloor z \rfloor _{+} := \max (z,0),\ z \in \mathbb {R} \end{aligned}$$

Using the substitution $v:=u^{1/m}$, we have the second variant

96 $$\begin{aligned} \partial _t{v} - \nabla \cdot (m|v|^{m-1}\nabla v) = 0, \qquad m>1, \end{aligned}$$

having the solution

97 $$\begin{aligned} v(x_1,x_2,t) = \bigg \{ \frac{1}{1+t} \bigg ( \bigg \lfloor 1 - \frac{m-1}{4m^2} \frac{x_1^2+x_2^2}{(1+t)^{1/m}} \bigg \rfloor _{+}\bigg )^{ \frac{m}{m-1} } \bigg \}^{1/m}. \end{aligned}$$

For both problems ((94)–(95) and (96)–(97)), the computational domain is $\varOmega = (-6, 6)^2$ and the Dirichlet boundary condition is prescribed on all boundaries by (95) or (97). The final time is $T=1$.

We carried out computation using a sequence of uniform triangular grids (having 288, 1152, 4608 and 18432 triangles) with several combinations of polynomial approximation degrees with respect to space (*p*) and time (*q*). The time step has been chosen constant $\tau =0.01$. Besides the error quantities ($\widetilde{{{\mathcal {R}}}}({u_h^{\tau }})$ and ${{\mathcal {J}}}({u_h^{\tau }})$) and its estimators $\eta$, $\eta _{R}:=\sum _{K,m}\eta _{R,K,m}$, $\eta _{S}:=\sum _{K,m}\eta _{S,K,m}$ and $\eta _{T}:=\sum _{K,m}\eta _{T,K,m}$, we evaluate the effectivity indices

98 $$\begin{aligned} i_{\textrm{eff}}= \frac{\eta }{\widetilde{{{\mathcal {R}}}}({u_h^{\tau }})}, \qquad i_{\textrm{eff}}^{\textrm{tot}}= \frac{ \left( \eta ^2 + {{\mathcal {J}}}({u_h^{\tau }})\right) ^{1/2}}{\widetilde{{{\mathcal {E}}}}({u_h^{\tau }})}. \end{aligned}$$

In addition, we present the experimental order of convergence (EoC) of the errors and the estimators for each pair of successive meshes.

Tables 1–4 show the results for both Barenblatt problems ((94)–(95) with $m=0.25$ and (96)–(97) with $m=2$) with two variants of the scaling parameter ${d_{K,m}}$, $K\in {{\mathcal {T}}_h^m}$, $m=1,\dots ,r$ given by (21a) and (21b). The quantity $\#\textrm{DoF}$ represents the number of degrees of freedom in the space, that is, $\#\textrm{DoF}=\dim {S_{hp,m}}$, $m=1,\dots ,r$. We observe a good correspondence between $\widetilde{{{\mathcal {R}}}}({u_h^{\tau }})$ and $\eta$, the effectivity index $i_{\textrm{eff}}$ varies between 1 and 2.5 for all tested values of *p* and *q* and both variants of ${d_{K,m}}$ ((21a) and (21b)).

**Table 1:** Barenblatt problem (94)–(95), $m=0.25$, scaling parameter ${d_{K,m}}$ given by (21a), approximation of the error and the error estimators, EOC in parenthesis

*h*	$\#\textrm{DoF}$	$\widetilde{{{\mathcal {R}}}}({u_h^{\tau }})$	$\eta$	${{\mathcal {J}}}({u_h^{\tau }})$	$\eta _R$	$\eta _S$	$\eta _T$	$i_{\textrm{eff}}$	$i_{\textrm{eff}}^{\textrm{tot}}$
$\hbox {p}=1$ & $\hbox {q}=1$									
1.41	864	$8.42 \times 10^{-4}$	$1.46 \times 10^{-3}$	$4.01 \times 10^{-3}$	$7.67 \times 10^{-6}$	$1.22 \times 10^{-3}$	$7.94 \times 10^{-4}$	1.73	1.13
0.71	3456	$7.31 \times 10^{-4}$	$1.29 \times 10^{-3}$	$3.69 \times 10^{-3}$	$7.68 \times 10^{-6}$	$1.16 \times 10^{-3}$	$5.38 \times 10^{-4}$	1.76	1.13
		(0.20)	(0.18)	(0.12)	(0.00)	(0.07)	(0.56)		
0.35	13824	$4.98 \times 10^{-4}$	$1.04 \times 10^{-3}$	$2.95 \times 10^{-3}$	$8.56 \times 10^{-6}$	$1.02 \times 10^{-3}$	$1.55 \times 10^{-4}$	2.09	1.16
		(0.55)	(0.30)	(0.33)	($-$0.16)	(0.18)	(1.80)		
0.18	55296	$4.40 \times 10^{-4}$	$1.01 \times 10^{-3}$	$2.81 \times 10^{-3}$	$9.00 \times 10^{-6}$	$1.01 \times 10^{-3}$	$3.22 \times 10^{-5}$	2.31	1.18
		(0.18)	(0.04)	(0.07)	($-$0.07)	(0.02)	(2.26)		
$\hbox {p}=2$ & $\hbox {q}=2$									
1.41	1728	$2.06 \times 10^{-4}$	$3.49 \times 10^{-4}$	$1.32 \times 10^{-3}$	$5.60 \times 10^{-7}$	$3.17 \times 10^{-4}$	$1.46 \times 10^{-4}$	1.70	1.09
0.71	6912	$1.16 \times 10^{-4}$	$1.86 \times 10^{-4}$	$7.91 \times 10^{-4}$	$4.59 \times 10^{-8}$	$1.74 \times 10^{-4}$	$6.64 \times 10^{-5}$	1.60	1.08
		(0.82)	(0.91)	(0.74)	(3.61)	(0.86)	(1.14)		
0.35	27648	$6.51 \times 10^{-5}$	$8.69 \times 10^{-5}$	$4.33 \times 10^{-4}$	$5.46 \times 10^{-8}$	$8.57 \times 10^{-5}$	$1.44 \times 10^{-5}$	1.34	1.04
		(0.84)	(1.10)	(0.87)	($-$0.25)	(1.02)	(2.20)		
0.18	110592	$3.51 \times 10^{-5}$	$4.34 \times 10^{-5}$	$2.28 \times 10^{-4}$	$6.54 \times 10^{-8}$	$4.32 \times 10^{-5}$	$2.46 \times 10^{-6}$	1.23	1.03
		(0.89)	(1.00)	(0.92)	($-$0.26)	(0.99)	(2.56)		
$\hbox {p}=3$ & $\hbox {q}=2$									
1.41	2880	$6.32 \times 10^{-5}$	$1.16 \times 10^{-4}$	$3.74 \times 10^{-4}$	$4.39 \times 10^{-8}$	$9.31 \times 10^{-5}$	$6.83 \times 10^{-5}$	1.83	1.12
0.71	11520	$2.03 \times 10^{-5}$	$3.02 \times 10^{-5}$	$1.45 \times 10^{-4}$	$3.48 \times 10^{-8}$	$2.82 \times 10^{-5}$	$1.08 \times 10^{-5}$	1.49	1.06
		(1.64)	(1.94)	(1.37)	(0.33)	(1.73)	(2.66)		
0.35	46080	$5.77 \times 10^{-6}$	$8.16 \times 10^{-6}$	$4.11 \times 10^{-5}$	$5.31 \times 10^{-8}$	$8.03 \times 10^{-6}$	$1.20 \times 10^{-6}$	1.41	1.05
		(1.81)	(1.89)	(1.81)	($-$0.61)	(1.81)	(3.17)		
0.18	184320	$1.35 \times 10^{-6}$	$2.09 \times 10^{-6}$	$9.47 \times 10^{-6}$	$6.53 \times 10^{-8}$	$2.04 \times 10^{-6}$	$8.52 \times 10^{-8}$	1.55	1.07
		(2.10)	(1.97)	(2.12)	($-$0.30)	(1.98)	(3.82)

**Table 2:** Barenblatt problem (94)–(95), $m=0.25$, scaling parameter ${d_{K,m}}$ given by (21b), approximation of the error and the error estimators, EOC in parenthesis

*h*	$\#\textrm{DoF}$	$\widetilde{{{\mathcal {R}}}}({u_h^{\tau }})$	$\eta$	${{\mathcal {J}}}({u_h^{\tau }})$	$\eta _R$	$\eta _S$	$\eta _T$	$i_{\textrm{eff}}$	$i_{\textrm{eff}}^{\textrm{tot}}$
$\hbox {p}=1$ & $\hbox {q}=1$									
1.41	864	$2.34 \times 10^{-1}$	$4.06 \times 10^{-1}$	1.12	$2.14 \times 10^{-3}$	$3.39 \times 10^{-1}$	$2.21 \times 10^{-1}$	1.73	1.13
0.71	3456	$1.02 \times 10^{-1}$	$1.79 \times 10^{-1}$	$5.14 \times 10^{-1}$	$1.07 \times 10^{-3}$	$1.62 \times 10^{-1}$	$7.50 \times 10^{-2}$	1.76	1.13
		(1.20)	(1.18)	(1.12)	(1.00)	(1.07)	(1.56)		
0.35	13824	$3.47 \times 10^{-2}$	$7.26 \times 10^{-2}$	$2.05 \times 10^{-1}$	$5.96 \times 10^{-4}$	$7.14 \times 10^{-2}$	$1.08 \times 10^{-2}$	2.09	1.16
		(1.55)	(1.30)	(1.33)	(0.84)	(1.18)	(2.80)		
0.18	55296	$1.53 \times 10^{-2}$	$3.54 \times 10^{-2}$	$9.80 \times 10^{-2}$	$3.14 \times 10^{-4}$	$3.51 \times 10^{-2}$	$1.12 \times 10^{-3}$	2.31	1.18
		(1.18)	(1.04)	(1.07)	(0.93)	(1.02)	(3.26)		
0.09	221184	$7.63 \times 10^{-3}$	$1.76 \times 10^{-2}$	$4.85 \times 10^{-2}$	$1.59 \times 10^{-4}$	$1.75 \times 10^{-2}$	$1.44 \times 10^{-4}$	2.31	1.18
		(1.01)	(1.00)	(1.01)	(0.98)	(1.00)	(2.97)		
$\hbox {p}=2$ & $\hbox {q}=2$									
1.41	1728	$5.73 \times 10^{-2}$	$9.73 \times 10^{-2}$	$3.68 \times 10^{-1}$	$1.56 \times 10^{-4}$	$8.83 \times 10^{-2}$	$4.08 \times 10^{-2}$	1.70	1.09
0.71	6912	$1.62 \times 10^{-2}$	$2.60 \times 10^{-2}$	$1.10 \times 10^{-1}$	$6.40 \times 10^{-6}$	$2.43 \times 10^{-2}$	$9.24 \times 10^{-3}$	1.60	1.08
		(1.82)	(1.91)	(1.74)	(4.61)	(1.86)	(2.14)		
0.35	27648	$4.53 \times 10^{-3}$	$6.06 \times 10^{-3}$	$3.02 \times 10^{-2}$	$3.81 \times 10^{-6}$	$5.97 \times 10^{-3}$	$1.01 \times 10^{-3}$	1.34	1.04
		(1.84)	(2.10)	(1.87)	(0.75)	(2.02)	(3.20)		
0.18	110292	$1.22 \times 10^{-3}$	$1.51 \times 10^{-3}$	$7.96 \times 10^{-3}$	$2.28 \times 10^{-6}$	$1.51 \times 10^{-3}$	$8.56 \times 10^{-5}$	1.23	1.03
		(1.89)	(2.00)	(1.92)	(0.74)	(1.99)	(3.55)		
$\hbox {p}=3$ & $\hbox {q}=2$									
1.41	2880	$1.76 \times 10^{-2}$	$3.22 \times 10^{-2}$	$1.04 \times 10^{-1}$	$1.22 \times 10^{-5}$	$2.59 \times 10^{-2}$	$1.90 \times 10^{-2}$	1.83	1.12
0.71	11520	$2.83 \times 10^{-3}$	$4.20 \times 10^{-3}$	$2.02 \times 10^{-2}$	$4.85 \times 10^{-6}$	$3.92 \times 10^{-3}$	$1.51 \times 10^{-3}$	1.49	1.06
		(2.64)	(2.94)	(2.37)	(1.33)	(2.73)	(3.66)		
0.35	46080	$4.42 \times 10^{-4}$	$5.68 \times 10^{-4}$	$2.87 \times 10^{-3}$	$3.70 \times 10^{-6}$	$5.59 \times 10^{-4}$	$8.38 \times 10^{-5}$	1.29	1.04
		(2.68)	(2.89)	(2.81)	(0.39)	(2.81)	(4.17)		
0.18	184320	$4.71 \times 10^{-5}$	$7.28 \times 10^{-5}$	$3.30 \times 10^{-4}$	$2.27 \times 10^{-6}$	$7.10 \times 10^{-5}$	$2.97 \times 10^{-6}$	1.55	1.07
		(3.23)	(2.97)	(3.12)	(0.70)	(2.98)	(4.82)

**Table 3:** Barenblatt problem (96)–(97), $m=2$, scaling parameter ${d_{K,m}}$ given by (21a), approximation of the error and the error estimators, EOC in parenthesis

*h*	$\#\textrm{DoF}$	$\widetilde{{{\mathcal {R}}}}({u_h^{\tau }})$	$\eta$	${{\mathcal {J}}}({u_h^{\tau }})$	$\eta _R$	$\eta _S$	$\eta _T$	$i_{\textrm{eff}}$	$i_{\textrm{eff}}^{\textrm{tot}}$
$\hbox {p}=1$ & $\hbox {q}=1$									
1.41	864	$3.35 \times 10^{-3}$	$5.76 \times 10^{-3}$	$1.59 \times 10^{-2}$	$6.44 \times 10^{-7}$	$4.58 \times 10^{-3}$	$3.50 \times 10^{-3}$	1.72	1.13
0.71	3456	$1.71 \times 10^{-3}$	$3.19 \times 10^{-3}$	$7.67 \times 10^{-3}$	$5.90 \times 10^{-7}$	$2.74 \times 10^{-3}$	$1.64 \times 10^{-3}$	1.87	1.16
		(0.97)	(0.85)	(1.05)	(0.13)	(0.74)	(1.09)		
0.35	13824	$1.09 \times 10^{-3}$	$2.27 \times 10^{-3}$	$4.57 \times 10^{-3}$	$4.81 \times 10^{-7}$	$2.17 \times 10^{-3}$	$6.69 \times 10^{-4}$	2.07	1.21
		(0.64)	(0.49)	(0.75)	(0.29)	(0.34)	(1.29)		
0.18	55296	$9.17 \times 10^{-4}$	$2.04 \times 10^{-3}$	$3.60 \times 10^{-3}$	$3.62 \times 10^{-7}$	$2.02 \times 10^{-3}$	$2.41 \times 10^{-4}$	2.22	1.25
		(0.26)	(0.15)	(0.34)	(0.41)	(0.10)	(1.48)		
$\hbox {p}=2$ & $\hbox {q}=2$									
1.41	1728	$6.33 \times 10^{-4}$	$1.27 \times 10^{-3}$	$4.52 \times 10^{-3}$	$1.98 \times 10^{-7}$	$9.68 \times 10^{-4}$	$8.18 \times 10^{-4}$	2.00	1.12
0.71	6912	$3.26 \times 10^{-4}$	$5.98 \times 10^{-4}$	$2.20 \times 10^{-3}$	$1.63 \times 10^{-7}$	$4.70 \times 10^{-4}$	$3.71 \times 10^{-4}$	1.84	1.11
		(0.96)	(1.08)	(1.04)	(0.28)	(1.04)	(1.14)		
0.35	27648	$1.91 \times 10^{-4}$	$2.99 \times 10^{-4}$	$1.17 \times 10^{-3}$	$1.24 \times 10^{-7}$	$2.60 \times 10^{-4}$	$1.47 \times 10^{-4}$	1.57	1.08
		(0.77)	(1.00)	(0.91)	(0.40)	(0.85)	(1.33)		
0.18	110592	$1.23 \times 10^{-4}$	$1.71 \times 10^{-4}$	$7.18 \times 10^{-4}$	$8.96 \times 10^{-8}$	$1.63 \times 10^{-4}$	$5.26 \times 10^{-5}$	1.39	1.06
		(0.63)	(0.81)	(0.71)	(0.47)	(0.68)	(1.48)		
$\hbox {p}=3$ & $\hbox {q}=2$									
1.41	2880	$2.57 \times 10^{-4}$	$5.65 \times 10^{-4}$	$2.00 \times 10^{-3}$	$1.26 \times 10^{-7}$	$3.69 \times 10^{-4}$	$4.28 \times 10^{-4}$	2.20	1.14
0.71	11520	$1.51 \times 10^{-4}$	$2.70 \times 10^{-4}$	$1.06 \times 10^{-3}$	$8.66 \times 10^{-8}$	$2.07 \times 10^{-4}$	$1.74 \times 10^{-4}$	1.79	1.10
		(0.77)	(1.06)	(0.91)	(0.54)	(0.84)	(1.30)		
0.35	46080	$1.00 \times 10^{-4}$	$1.50 \times 10^{-4}$	$6.70 \times 10^{-4}$	$6.12 \times 10^{-8}$	$1.33 \times 10^{-4}$	$6.91 \times 10^{-5}$	1.50	1.06
		(0.59)	(0.85)	(0.66)	(0.50)	(0.63)	(1.33)		
0.18	184320	$6.92 \times 10^{-5}$	$9.37 \times 10^{-5}$	$4.48 \times 10^{-4}$	$4.30 \times 10^{-8}$	$9.04 \times 10^{-5}$	$2.46 \times 10^{-5}$	1.35	1.05
		(0.53)	(0.68)	(0.58)	(0.51)	(0.56)	(1.49)

**Table 4:** Barenblatt problem (96)–(97), $m=2$, scaling parameter ${d_{K,m}}$ given by (21b), approximation of the error and the error estimators, EOC in parenthesis

*h*	$\#\textrm{DoF}$	$\widetilde{{{\mathcal {R}}}}({u_h^{\tau }})$	$\eta$	${{\mathcal {J}}}({u_h^{\tau }})$	$\eta _R$	$\eta _S$	$\eta _T$	$i_{\textrm{eff}}$	$i_{\textrm{eff}}^{\textrm{tot}}$
$\hbox {p}=1$ & $\hbox {q}=1$									
1.41	864	$3.35 \times 10^{-1}$	$5.76 \times 10^{-1}$	1.59	$6.44 \times 10^{-5}$	$4.58 \times 10^{-1}$	$3.50 \times 10^{-1}$	1.72	1.13
0.71	3456	$8.54 \times 10^{-2}$	$1.60 \times 10^{-1}$	$3.84 \times 10^{-1}$	$2.95 \times 10^{-5}$	$1.37 \times 10^{-1}$	$8.19 \times 10^{-2}$	1.87	1.16
		(1.97)	(1.85)	(2.05)	(1.13)	(1.74)	(2.09)		
0.35	13824	$2.74 \times 10^{-2}$	$5.68 \times 10^{-2}$	$1.14 \times 10^{-1}$	$1.20 \times 10^{-5}$	$5.43 \times 10^{-2}$	$1.68 \times 10^{-2}$	2.07	1.21
		(1.64)	(1.49)	(1.75)	(1.29)	(1.34)	(2.29)		
0.18	55296	$1.15 \times 10^{-2}$	$2.57 \times 10^{-2}$	$4.53 \times 10^{-2}$	$4.56 \times 10^{-6}$	$2.55 \times 10^{-2}$	$3.03 \times 10^{-3}$	2.22	1.25
		(1.25)	(1.15)	(1.34)	(1.40)	(1.09)	(2.47)		
0.09	221184	$5.54 \times 10^{-3}$	$1.27 \times 10^{-2}$	$2.13 \times 10^{-2}$	$1.64 \times 10^{-6}$	$1.26 \times 10^{-2}$	$6.05 \times 10^{-4}$	2.29	1.27
		(1.06)	(1.02)	(1.09)	(1.48)	(1.01)	(2.32)		
$\hbox {p}=2$ & $\hbox {q}=2$									
1.41	1728	$6.33 \times 10^{-2}$	$1.27 \times 10^{-1}$	$4.52 \times 10^{-1}$	$1.98 \times 10^{-5}$	$9.68 \times 10^{-2}$	$8.18 \times 10^{-2}$	2.00	1.12
0.71	6912	$1.63 \times 10^{-2}$	$2.99 \times 10^{-2}$	$1.10 \times 10^{-1}$	$8.16 \times 10^{-6}$	$2.35 \times 10^{-2}$	$1.85 \times 10^{-2}$	1.84	1.11
		(1.96)	(2.08)	(2.04)	(1.28)	(2.04)	(2.14)		
0.35	27648	$4.78 \times 10^{-3}$	$7.49 \times 10^{-3}$	$2.94 \times 10^{-2}$	$3.10 \times 10^{-6}$	$6.52 \times 10^{-3}$	$3.69 \times 10^{-3}$	1.57	1.08
		(1.77)	(2.00)	(1.90)	(1.40)	(1.85)	(2.33)		
0.18	110592	$1.55 \times 10^{-3}$	$2.15 \times 10^{-3}$	$9.04 \times 10^{-3}$	$1.13 \times 10^{-6}$	$2.04 \times 10^{-3}$	$6.62 \times 10^{-4}$	1.39	1.06
		(1.63)	(1.80)	(1.70)	(1.46)	(1.67)	(2.48)		
$\hbox {p}=3$ & $\hbox {q}=2$									
1.41	2880	$2.57 \times 10^{-2}$	$5.65 \times 10^{-2}$	$2.00 \times 10^{-1}$	$1.26 \times 10^{-5}$	$3.69 \times 10^{-2}$	$4.28 \times 10^{-2}$	2.20	1.14
0.71	11520	$7.54 \times 10^{-3}$	$1.35 \times 10^{-2}$	$5.31 \times 10^{-2}$	$4.33 \times 10^{-6}$	$1.03 \times 10^{-2}$	$8.70 \times 10^{-3}$	1.79	1.10
		(1.77)	(2.06)	(1.91)	(1.54)	(1.84)	(2.30)		
0.35	46080	$2.51 \times 10^{-3}$	$3.76 \times 10^{-3}$	$1.68 \times 10^{-2}$	$1.53 \times 10^{-6}$	$3.34 \times 10^{-3}$	$1.73 \times 10^{-3}$	1.50	1.06
		(1.59)	(1.85)	(1.66)	(1.50)	(1.63)	(2.33)		
0.18	184320	$8.71 \times 10^{-4}$	$1.18 \times 10^{-3}$	$5.64 \times 10^{-3}$	$5.42 \times 10^{-7}$	$1.14 \times 10^{-3}$	$3.09 \times 10^{-4}$	1.36	1.05
		(1.53)	(1.67)	(1.57)	(1.50)	(1.55)	(2.48)		

Finally, we note that the experimental orders of convergence EoC in Tables 1–4) of the error $\widetilde{{{\mathcal {R}}}}({u_h^{\tau }})$ and its estimate $\eta$ are $O(h^p)$ for the choice (21b) of the scaling parameter ${d_{K,m}}$ but only $O(h^{p-1})$ for the choice (21a). This follows from the fact that $\tau _m \ll h_K$ for the computations of the Barenblatt problem and then the dominant part of ${d_{K,m}}$ is $\tau _m^{-2} T {\left\| \tfrac{{\mathrm d}{\vartheta }}{{\mathrm d}u}\right\| }_{K,m,\infty }$, cf. (21a), which implies that ${d_{K,m}}= O(h^0)$ (the time step is the same for all computations). The dominant part of the error estimator is $\eta _{S,K,m}$, hence if ${\left\| {\sigma _h^{\tau }}- {\sigma }({u_h^{\tau }}, \nabla {u_h^{\tau }}) \right\| }_{K,m} = O(h^{p})$ then $\eta _{S,K,m}= O(h^{p-1})$, cf. (42b). Nevertheless, comparing the pairs of Tables 1–2 and Tables 3–4, we found that the effectivity indexes are practically independent of the choice of ${d_{K,m}}$.

#### Justification of the Algebraic Stopping Criterion (91)

We present the numerical study of the stopping criterion (91) which is used in the iterative solution of algebraic systems given by (13). We consider again the Barenblatt problem (94)–(95) with $m=0.25$ and (96)–(97) with $m=2$. The user-dependent constant $c_A$ in (91) has been chosen as $10^{-1}$, $10^{-2}$, $10^{-3}$ and $10^{-4}$. Tables 5 and 6 show the estimators $\eta$, ${{\mathcal {J}}}({u_h^{\tau }})$, ${\eta _{\textrm{alg}}}$ and ${\eta _{\textrm{alg}}}$, cf. (90), for selected meshes and polynomial approximation degrees and the scaling parameter ${d_{K,m}}$ chosen by (21a).

Additionally, we present the total number of steps of the Newton-like solver ${N_{\textrm{non}}}$, the total number of GMRES iterations ${N_{\textrm{lin}}}$ and the computational time in seconds. The computational time has only an informative character.

**Table 5:** Barenblatt problem (94)–(95), $m=0.25$, scaling parameter ${d_{K,m}}$ given by (21a), numerical study of the algebraic stopping criterion (91)

$c_A$	$\eta$	${{\mathcal {J}}}({u_h^{\tau }})$	${\eta _{\textrm{alg}}}$	${\eta _{\textrm{spa}}}$	${N_{\textrm{non}}}$	${N_{\textrm{lin}}}$	time(s)
$$h= 0.35$$, $$ p= 1 $$ & $$q = 1 $$, $$\#\textrm{DoF}= 13824 $$							
$1.0 \times 10^{-1}$	$1.2475 \times 10^{-3}$	$3.0760 \times 10^{-3}$	$8.1322 \times 10^{-4}$	$1.3804 \times 10^{-2}$	202	14148	422.1
$1.0 \times 10^{-2}$	$1.0559 \times 10^{-3}$	$2.9565 \times 10^{-3}$	$7.6470 \times 10^{-5}$	$1.3483 \times 10^{-2}$	362	21589	606.8
$1.0 \times 10^{-3}$	$1.0435 \times 10^{-3}$	$2.9468 \times 10^{-3}$	$7.9268 \times 10^{-6}$	$1.3458 \times 10^{-2}$	529	26545	693.6
$1.0 \times 10^{-4}$	$1.0423 \times 10^{-3}$	$2.9457 \times 10^{-3}$	$7.3279 \times 10^{-7}$	$1.3456 \times 10^{-2}$	579	27766	705.1
$$h= 0.35$$, $$ p= 2 $$ & $$q = 2 $$, $$\#\textrm{DoF}= 27648 $$							
$1.0 \times 10^{-1}$	$1.0443 \times 10^{-4}$	$4.3586 \times 10^{-4}$	$5.7369 \times 10^{-5}$	$1.1019 \times 10^{-3}$	406	10581	1968.3
$1.0 \times 10^{-2}$	$8.8249 \times 10^{-5}$	$4.3375 \times 10^{-4}$	$6.1984 \times 10^{-6}$	$1.0961 \times 10^{-3}$	536	12059	2119.4
$1.0 \times 10^{-3}$	$8.7054 \times 10^{-5}$	$4.3350 \times 10^{-4}$	$6.0680 \times 10^{-7}$	$1.0956 \times 10^{-3}$	576	12541	2030.1
$1.0 \times 10^{-4}$	$8.6948 \times 10^{-5}$	$4.3347 \times 10^{-4}$	$5.1172 \times 10^{-8}$	$1.0955 \times 10^{-3}$	618	13580	2132.1
$$h= 0.35$$, $$ p= 3 $$ & $$q = 2 $$, $$\#\textrm{DoF}= 46080 $$							
$1.0 \times 10^{-1}$	$9.9098 \times 10^{-6}$	$4.1201 \times 10^{-5}$	$5.3825 \times 10^{-6}$	$1.0693 \times 10^{-4}$	534	10480	6610.2
$1.0 \times 10^{-2}$	$8.2946 \times 10^{-6}$	$4.1156 \times 10^{-5}$	$6.0342 \times 10^{-7}$	$1.0670 \times 10^{-4}$	602	11479	6998.3
$1.0 \times 10^{-3}$	$8.1647 \times 10^{-6}$	$4.1150 \times 10^{-5}$	$4.5285 \times 10^{-8}$	$1.0669 \times 10^{-4}$	636	12288	7181.7
$1.0 \times 10^{-4}$	$8.1577 \times 10^{-6}$	$4.1150 \times 10^{-5}$	$4.5439 \times 10^{-9}$	$1.0669 \times 10^{-4}$	668	13178	7566.5

**Table 6:** Barenblatt problem (96)–(97), $m=2$, scaling parameter ${d_{K,m}}$ given by (21a), numerical study of the algebraic stopping criterion (91)

$c_A$	$\eta$	${{\mathcal {J}}}({u_h^{\tau }})$	${\eta _{\textrm{alg}}}$	${\eta _{\textrm{spa}}}$	${N_{\textrm{non}}}$	${N_{\textrm{lin}}}$	time(s)
$$h= 0.35$$, $$ p= 1 $$ & $$q = 1 $$, $$\#\textrm{DoF}= 13824 $$							
$1.0 \times 10^{-1}$	$2.3055 \times 10^{-3}$	$4.6659 \times 10^{-3}$	$4.0757 \times 10^{-4}$	$9.3084 \times 10^{-3}$	100	2199	224.6
$1.0 \times 10^{-2}$	$2.2689 \times 10^{-3}$	$4.5703 \times 10^{-3}$	$1.3712 \times 10^{-5}$	$9.2211 \times 10^{-3}$	200	4957	284.5
$1.0 \times 10^{-3}$	$2.2688 \times 10^{-3}$	$4.5694 \times 10^{-3}$	$3.5878 \times 10^{-6}$	$9.2216 \times 10^{-3}$	299	7715	352.6
$1.0 \times 10^{-4}$	$2.2688 \times 10^{-3}$	$4.5696 \times 10^{-3}$	$3.9773 \times 10^{-7}$	$9.2220 \times 10^{-3}$	378	9790	413.7
$$h= 0.35$$, $$ p= 2 $$ & $$q = 2 $$, $$\#\textrm{DoF}= 27648 $$							
$1.0 \times 10^{-1}$	$3.0332 \times 10^{-4}$	$1.1855 \times 10^{-3}$	$7.0279 \times 10^{-5}$	$1.7702 \times 10^{-3}$	201	4065	1535.3
$1.0 \times 10^{-2}$	$2.9940 \times 10^{-4}$	$1.1753 \times 10^{-3}$	$7.2859 \times 10^{-6}$	$1.7634 \times 10^{-3}$	286	5820	1675.9
$1.0 \times 10^{-3}$	$2.9916 \times 10^{-4}$	$1.1747 \times 10^{-3}$	$8.0717 \times 10^{-7}$	$1.7619 \times 10^{-3}$	393	7803	1759.7
$1.0 \times 10^{-4}$	$2.9916 \times 10^{-4}$	$1.1747 \times 10^{-3}$	$8.9211 \times 10^{-8}$	$1.7620 \times 10^{-3}$	529	10172	1984.6
$$h= 0.35$$, $$ p= 3 $$ & $$q = 2 $$, $$\#\textrm{DoF}= 46080 $$							
$1.0 \times 10^{-1}$	$1.5523 \times 10^{-4}$	$6.8813 \times 10^{-4}$	$7.1710 \times 10^{-5}$	$1.1705 \times 10^{-3}$	202	4222	5539.6
$1.0 \times 10^{-2}$	$1.5037 \times 10^{-4}$	$6.6961 \times 10^{-4}$	$5.7493 \times 10^{-6}$	$1.1586 \times 10^{-3}$	316	6538	6068.2
$1.0 \times 10^{-3}$	$1.5026 \times 10^{-4}$	$6.6968 \times 10^{-4}$	$6.3387 \times 10^{-7}$	$1.1580 \times 10^{-3}$	453	8880	6615.7
$1.0 \times 10^{-4}$	$1.5026 \times 10^{-4}$	$6.6967 \times 10^{-4}$	$5.9895 \times 10^{-8}$	$1.1580 \times 10^{-3}$	591	11150	7101.3

We observe that the error estimators $\eta$, ${{\mathcal {J}}}({u_h^{\tau }})$ and also ${\eta _{\textrm{spa}}}$ converge to the limit values for decreasing $c_A$ in (91) which mimic the case when the algebraic errors are negligible. Moreover, the relative differences between the actual values $\eta$ and ${{\mathcal {J}}}({u_h^{\tau }})$ and their limits correspond more or less to the value of $c_A$. Obviously, smaller values of $c_A$ cause prolongation of the computational time, due to a higher number of iterations, with a negligible effect on accuracy. Thus, the choice $c_A=10^{-2}$ seems to be optimal in order to balance accuracy and efficiency.

The presented numerical experiments indicate that the estimator ${\eta _{\textrm{spa}}}({u_h^{\tau }})$ gives an upper bound of ${{\mathcal {R}}}({u_h^{\tau }})$, however, this observation is not supported by the theory. The quantity ${\eta _{\textrm{spa}}}({u_h^{\tau }})$ is used only in the stopping criterion (91).

### Tracy Problem

Tracy problem represents a standard benchmark, where the analytical solutions of the Richards equation are available [35]. We consider the Gardners constitutive relations [26]

99 $$\begin{aligned} {\textbf{K}}(u) = \left\{ \begin{array}{ll} {\textbf{K}}_s \exp (-\alpha \psi ) &  \quad \hbox { if}\ \psi>0\\ {\textbf{K}}_s &  \quad \hbox { if}\ \psi \le 0 \end{array} \right. ,\qquad {\vartheta }(u) = \left\{ \begin{array} {ll} \theta _r + (\theta _s - \theta _r) \exp (-\alpha \psi ) &  \quad \hbox { if}\ \psi >0\\ \theta _s &  \quad \hbox { if}\ \psi \le 0 \end{array} \right. \end{aligned}$$

where $\psi = u - z$ is the pressure head, *z* is the vertical coordinate and the material parameters ${\textbf{K}}_s=1.2{\mathbb {I}}$, $\theta _s=0.5$, $\theta _r=0.0$, and $\alpha =0.1$ are the isotropic conductivity, saturated water content, residual water content, and the soil index parameter related to the pore-size distribution, respectively.

The computational domain is ${\varOmega }=(0,1)^2$, the initial condition is set $u = u_r: = -10$ in ${\varOmega }$ where $u_r$ corresponds to the hydraulic head when the porous medium is dry. On the top part of the boundary ${\varGamma }_1:=\{(x, z),\ x\in (0,1),\ z= 1\}$, we prescribe the boundary condition

100 $$\begin{aligned} u(x) = \frac{1}{\alpha } \log \Big (\exp (\alpha u_r) + (1-\exp (\alpha u_r) \sin ( \pi x) \Big ),\qquad x\in (0,1) \end{aligned}$$

and on the rest of boundary ${\varGamma }$ we set $u = u_r$. We note that this benchmark poses an inconsistency between the initial and boundary conditions on ${\varGamma }_1$. Hence, the most challenging part is the computation close to $t=0$. In order to avoid the singularity at $t=0$, we investigate the error only on the interval $t\in [1.0 \times 10^{-5}, 1.1 \times 10^{-4}]$ with the fixed time step $\tau$ is $1.0 \times 10^{-6}$.

We perform a computation using a sequence of uniform triangular grids with several combinations of polynomial approximation degrees and the choice (21b), the results are shown in Table 7. We observe reasonable values of the effectivity indices except for the finest grids and the higher degrees of polynomial approximation, where the effectivity indices $i_{\textrm{eff}}$ are below 1. Based on the values of EoC, we suppose that $i_{\textrm{eff}}$ below 1 is not caused by the failure of the error estimator but due to an inaccurate approximation $\widetilde{{{\mathcal {R}}}}({u_h^{\tau }})$ of the exact error; see Remark 3.

**Table 7:** Tracy problem scaling parameter ${d_{K,m}}$ given by (21b), approximation of the error and the error estimators, EOC in parenthesis

*h*	$\#\textrm{DoF}$	$\widetilde{{{\mathcal {R}}}}({u_h^{\tau }})$	$\eta$	${{\mathcal {J}}}({u_h^{\tau }})$	$\eta _R$	$\eta _S$	$\eta _T$	$i_{\textrm{eff}}$	$i_{\textrm{eff}}^{\textrm{tot}}$
$\hbox {p}=1$ & $\hbox {q}=1$									
0.18	384	$2.43 \times 10^{-1}$	$2.95 \times 10^{-1}$	$6.62 \times 102$	$2.25 \times 10^{-3}$	$2.90 \times 10^{-1}$	$4.83 \times 10^{-2}$	1.22	1.00
0.09	1536	$8.77 \times 10^{-2}$	$1.19 \times 10^{-1}$	$2.47 \times 102$	$9.92 \times 10^{-4}$	$1.10 \times 10^{-1}$	$4.36 \times 10^{-2}$	1.35	1.00
		(1.47 )	(1.32)	(1.43)	(1.18)	(1.40)	(0.15)		
0.04	6144	$1.50 \times 10^{-2}$	$2.51 \times 10^{-2}$	$4.68 \times 101$	$1.33 \times 10^{-4}$	$2.39 \times 10^{-2}$	$7.31 \times 10^{-3}$	1.67	1.00
		(2.55)	(2.24)	(2.40)	(2.90)	(2.20)	(2.58)		
0.02	24576	$7.34 \times 10^{-3}$	$1.22 \times 10^{-2}$	$2.27 \times 101$	$8.11 \times 10^{-5}$	$1.20 \times 10^{-2}$	$1.86 \times 10^{-3}$	1.66	1.00
		(1.03)	(1.04)	(1.05)	(0.71)	(0.99)	(1.98)		
$\hbox {p}=2$ & $\hbox {q}=2$									
0.18	768	$4.88 \times 10^{-2}$	$6.18 \times 10^{-2}$	$1.92 \times 102$	$3.35 \times 10^{-4}$	$6.04 \times 10^{-2}$	$1.22 \times 10^{-2}$	1.27	1.00
0.09	3072	$1.75 \times 10^{-2}$	$1.98 \times 10^{-2}$	$6.03 \times 101$	$1.44 \times 10^{-5}$	$1.95 \times 10^{-2}$	$3.35 \times 10^{-3}$	1.13	1.00
		(1.48)	(1.65)	(1.67)	(4.54)	(1.63)	(1.86)		
0.04	12288	$5.59 \times 10^{-3}$	$6.28 \times 10^{-3}$	$1.94 \times 101$	$4.68 \times 10^{-6}$	$6.08 \times 10^{-3}$	$1.56 \times 10^{-3}$	1.12	1.00
		(1.64)	(1.65)	(1.64)	(1.62)	(1.68)	(1.11)		
0.02	49,152	$1.90 \times 10^{-3}$	$1.33 \times 10^{-3}$	4.37	$2.41 \times 10^{-6}$	$1.31 \times 10^{-3}$	$1.87 \times 10^{-4}$	0.70	1.00
		(1.56)	(2.24)	(2.15)	(0.96)	(2.21)	(3.06)		
$\hbox {p}=3$ & $\hbox {q}=2$									
0.18	1280	$2.24 \times 10^{-2}$	$2.64 \times 10^{-2}$	$9.60 \times 101$	$2.42 \times 10^{-5}$	$2.61 \times 10^{-2}$	$4.48 \times 10^{-3}$	1.18	1.00
0.09	5120	$6.26 \times 10^{-3}$	$7.94 \times 10^{-3}$	$2.77 \times 101$	$1.21 \times 10^{-5}$	$7.13 \times 10^{-3}$	$3.48 \times 10^{-3}$	1.27	1.00
		(1.84)	(1.74)	(1.79)	(1.00)	(1.87)	(0.37)		
0.04	20480	$1.40 \times 10^{-3}$	$4.60 \times 10^{-4}$	1.63	$2.87 \times 10^{-6}$	$4.49 \times 10^{-4}$	$8.90 \times 10^{-5}$	0.33	1.00
		(2.16)	(4.11)	(4.08)	(2.08)	(3.99)	(5.29)		
0.02	81920	$1.37 \times 10^{-3}$	$8.85 \times 10^{-5}$	$3.08 \times 10^{-1}$	$2.37 \times 10^{-6}$	$8.59 \times 10^{-5}$	$1.09 \times 10^{-5}$	0.06	1.00
		(0.03)	(2.38)	(2.41)	(0.28)	(2.39)	(3.03)		

## Mesh Adaptive Algorithm

We introduce the mesh adaptive algorithm which is based on the a posteriori error estimates $\eta$, cf. (41). Let $\delta >0$ be the given tolerance, the goal of the algorithm is to define the sequence of time steps $\tau _m$, meshes ${{\mathcal {T}}_h^m}$ and spaces ${S_{hp,m}}$, $m=1,\dots ,r$ such that the corresponding approximate solution ${u_h^{\tau }}\in {{S_{hp}^{\tau q}}}$ given by (13) satisfies the condition

101 $$\begin{aligned} \eta = \eta ({u_h^{\tau }}) \le \delta . \end{aligned}$$

Another possibility is to require $\left( \eta ^2 + {{\mathcal {J}}}({u_h^{\tau }})\right) ^{1/2} \le \delta$, then the following considerations have to be modified appropriately.

The mesh adaptation strategy is built on the equi-distribution principle, namely the sequences $\{\tau _m,\, {{\mathcal {T}}_h^m},\, {S_{hp,m}}\}_{m=1}^r$ should be generated such that

102a $$\begin{aligned} \eta _m&\le \delta _m:= \delta \sqrt{\tau _m/ T} \qquad \forall m=1,\dots ,r, \end{aligned}$$

102b $$\begin{aligned} \eta _{K,m}&\le \delta _{K,m}:= \delta _m \sqrt{1/\#{{\mathcal {T}}_h^m}} \qquad \forall K\in {{\mathcal {T}}_h^m}\ \forall m=1,\dots ,r, \end{aligned}$$

where $\eta _m:= (\sum _{K\in {{\mathcal {T}}_h^m}} \eta _{K,m}^2 )^{1/2}$ is the error estimate corresponding to the time interval $I_m$, $m=1,\dots ,r$ and $\#{{\mathcal {T}}_h^m}$ denotes the number of elements of ${{\mathcal {T}}_h^m}$. Obviously, if all the conditions in (102) are valid, then the criterion (101) is achieved.

**Algorithm 1:**
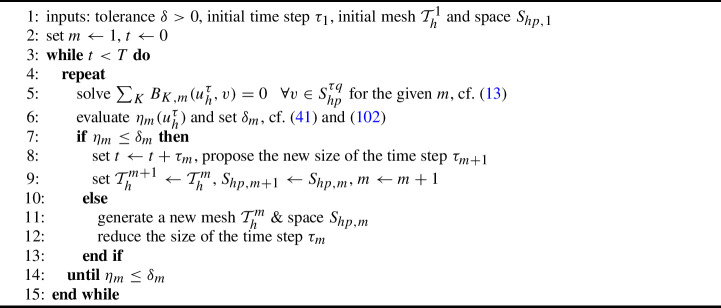
Space-time mesh adaptive algorithm.

Based on (101)–(102), we introduce the abstract Algorithm 1. The size of $\tau _m$, $m=1,\dots ,r$ (step 8 of the algorithm) are chosen to equilibrate estimates of the spatial and temporal reconstruction, $\eta _{S,m}:= (\sum _{K\in {{\mathcal {T}}_h^m}} (\eta _{S,K,m})^2)^{1/2}$ and $\eta _{T,m}:= (\sum _{K\in {{\mathcal {T}}_h^m}} (\eta _{T,K,m})^2)^{1/2}$, cf. (42). Particularly, we set the new time step according to the formula

103 $$\begin{aligned} \tau _{m+1} = \tau _m c_F \left( \frac{\eta _{S,m}}{\eta _{T,m}}\right) ^{1/(q+1)},\qquad m=1,\dots ,r, \end{aligned}$$

where $c_F\in (0,1)$ is the security factor and $q\ge 0$ is the polynomial degree with respect to time. Therefore, $q+1$ corresponds to the temporal order of convergence.

The construction of the new mesh (step 11 in Algorithm 1) is based on the modification of the anisotropic *hp*-mesh adaptation method from [15, 20]. Having the actual mesh ${{\mathcal {T}}_h^m}$, for each $K\in {{\mathcal {T}}_h^m}$ we set the new volume of *K* according the formula

104 $$\begin{aligned} \nu _K = |K| \varLambda (\delta _{K,m}/\eta _{K,m}),\qquad K\in {{\mathcal {T}}_h^m}, \end{aligned}$$

where $\delta _{K,m}$ is the local tolerance from (102b), |*K*| is the volume of |*K*| and $\varLambda :{\mathbb {R}}^+\rightarrow {\mathbb {R}}+$ is a suitable increasing function such that $\varLambda (1) = 1$. For particular variants of $\varLambda$, we refer to [15, 20].

When the new volume of mesh elements is established by (104), the new shape of *K* and a new polynomial approximation degree $p_K$ are optimized by minimizing the interpolation error. This optimization is done locally for each mesh element. In one adaptation level, we admit the increase or decrease of $p_K$ by one. Setting the new area, shape, and polynomial approximation degree for each element of the current mesh, we define the continuous mesh model [16] and carry out a remeshing using the code ANGENER [9].

The generated meshes are completely non-nested and non-matching, hence the evaluation of the time-penalty term (cf. Remark 1) is delicate. We refer to [20] where this aspect is described in detail and numerically verified. The presented numerical analysis takes into account the errors arising from the re-meshing in the temporal reconstruction ${R_h^{\tau }}$, which contains term $\big \{{{\vartheta }({u_h^{\tau }})}\big \}_{m-1}$, cf. (26). The following numerical experiments show that the error estimator is under the control also after each remeshing.

### Barenblatt Problem

We apply Algorithm 1 to the Barenblatt problem (96) with $m=2$. Table 8 shows the error estimators obtained by adaptive computation for three different tolerances $\delta$. Compared with the error estimators from Table 4, we observe that the adaptive computations achieve significantly smaller error estimates using a significantly smaller number of degrees of freedom. We note that we are not able to present the quantity $\widetilde{{{\mathcal {R}}}}$ (cf. (92)–(93)) approximating the error since the finite element code used for the evaluation of $\widetilde{{{\mathcal {R}}}}$ supports only uniform grids.

**Table 8:** Barenblatt problem (96)–(97), scaling parameter ${d_{K,m}}$ given by (21b), the error estimators obtained by the adaptive computations using Algorithm 1

*hp* adaptation						
$\delta$	$\#\textrm{DoF}$	$\eta$	${{\mathcal {J}}}({u_h^{\tau }})^{1/2}$	$\eta _{R}$	$\eta _S$	$\eta _{T}$
2.0E−03	4 543	$7.82 \times 10^{-4}$	$1.69 \times 10^{-3}$	$2.18 \times 10^{-4}$	$5.40 \times 10^{-4}$	$3.23 \times 10^{-4}$
1.0E−03	6 244	$4.57 \times 10^{-4}$	$1.17 \times 10^{-3}$	$1.48 \times 10^{-4}$	$3.13 \times 10^{-4}$	$1.43 \times 10^{-4}$
5.0E−04	9 071	$2.10 \times 10^{-4}$	$7.02 \times 10^{-4}$	$6.75 \times 10^{-5}$	$1.38 \times 10^{-4}$	$7.79 \times 10^{-5}$

The quantity $\#\textrm{DoF}$ is the average number of space degrees of freedom per one time step

**Fig. 1:**
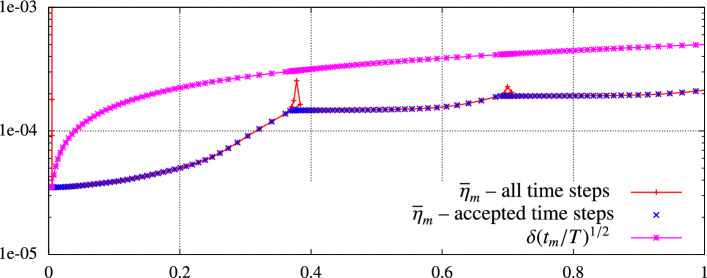
Barenblatt problem, (96)–(97), $m=2$, performance of Algorithm 1, accumulated error estimator ${\overline{\eta }}_m$ and the “accumulated” tolerance $\delta (t_m/T)^{1/2}$ for $m=1,\dots ,r$

Figure 1 shows the performance of Algorithm 1, where each dot corresponds to one time step $m=1,\dots ,r$. We plot the values of the accumulated estimators ${\overline{\eta }}_m =\sum _{i=1}^m \eta _i$ for all $m=1,\dots ,r$. The red nodes correspond to all computed time steps, including the rejected ones (steps 11–12 of Algorithm 1) whereas the blue nodes mark only the accepted time steps. The rejected time steps indicate the re-meshing. Moreover, we plot the “accumulated” tolerance $\delta (t_m/T)^{1/2}$, cf. (101) and (102a). We observe that the resulting estimator $\eta$ at $t=T$ is below the tolerance $\delta$ by a factor of approximately 2.5 since conditions (102) are stronger than (101).

**Fig. 2:**
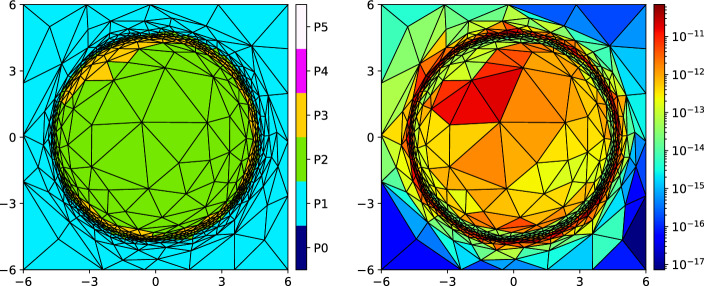
Barenblatt problem, *hp*-mesh obtained by Algorithm 1 (left) and the error estimators $\eta _{K,m}$, $K\in {{\mathcal {T}}_h^m}$ at $T=1$

Figure 2, left, shows the *hp*-mesh obtained by Algorithm 1 at the final time $T=1$, each triangle is highlighted by a color corresponding to the polynomial degree used $p_K$, $K\in {{\mathcal {T}}_h^m}$. We observe a strong anisotropic refinement about the circular singularity of the solution when $u\rightarrow 0^+$, see the analytical formula (97). Outside of this circle, large triangles with the smallest polynomial degree ($p=1$) are generated. On the other hand, due to the regularity of the solution in the interior of the circle, the polynomial degrees $p=2$ or $p=3$ are generated.

Moreover, Fig. 2, right, shows the error estimator $\eta _{K,m}$, $K\in {{\mathcal {T}}_h^m}$ at $T=1$. The elements in the exterior of the circle have small values of $\eta _{K,m}\approx 10^{-17}$ – $10^{-14}$ due to a constant solution and negligible errors. On the other hand, the values of $\eta _{K,m}$ for the rest of elements $K\in {{\mathcal {T}}_h^m}$ are in the range $10^{-13}$–$10^{-11}$ due to the equidistant principle used.

### Single Ring Infiltration

We deal with the numerical solution of the single ring infiltration experiment, which is frequently used for the identification of saturated hydraulic conductivity, cf. [32, 39] for example. We consider the Richards equation (3) where the active pore volume $\vartheta$ is given by (2), the water content function $\theta$ is given by the van Genuchten’s law [27] and the conductivity ${\textbf{K}}(u) ={\textbf{K}}_s {{{\mathcal {K}}}}_r(u)$ is given by the Mualem function [31], namely

105 $$\begin{aligned} \theta (u) = \left\{ \begin{array}{ll} \tfrac{\theta _s- \theta _r}{(1 + \left( -\alpha \psi )^n\right) ^{m}} + \theta _r &  \quad \text{ for } \psi< 0, \\ \theta _s &  \quad \text{ for } \psi \ge 0, \\ \end{array} \right. \nonumber \\ {{{\mathcal {K}}}}_r(u)&= \left\{ \begin{array}{ll} \tfrac{\left( 1-(-\alpha \psi )^{m\,n}(1 + (-\alpha \psi )^n)^{-m}\right) ^2}{\left( 1+(-\alpha \psi )^n\right) ^{m/2} } & \quad \text{ for } \psi < 0, \\ 1 &  \quad \text{ for } \psi \ge 0, \\ \end{array} \right. \end{aligned}$$

where $\psi = u - z$ is the pressure head, *z* is the vertical coordinate and the material parameters ${\textbf{K}}_s=0.048\,{\mathbb {I}}\,\,\mathrm {m\cdot hours^{-1}}$, $\theta _s=0.55$, $\theta _r=0.0$, $\alpha =0.8\,\mathrm {m^{-1}}$, $n=1.2$, $m=1/6$ and $S_s=10^{-3}\,\mathrm {m^{-1}}$ (cf. (2)).

**Fig. 3:**
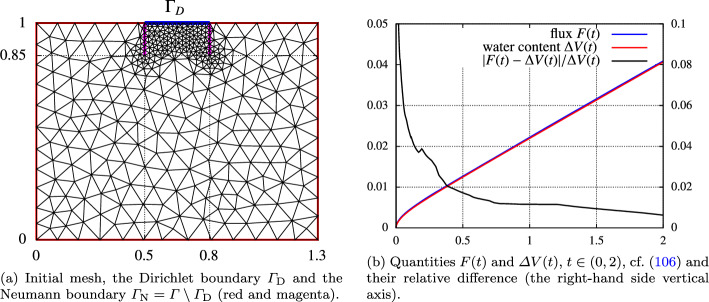
Single ring infiltration problem

The computational domain together with the boundary parts is sketched in Fig. 3a. On the boundary part $\varGamma _D$ we set the Dirichlet boundary condition $u= 1.05\,\textrm{m}$, and on ${\varGamma _\textrm{N}}={\varGamma }{\setminus }{\varGamma _\textrm{D}}$ we consider the homogeneous Neumann boundary condition. The smaller “magenta” vertical lines starting at ${\varGamma _\textrm{D}}$ belong to ${\varGamma _\textrm{N}}$. At $t=0$, a dry medium with $u= \psi + z =-2\,\textrm{m}$ is prescribed. We carried out the computation until the physical time $T=2\,\textrm{hours}$. The inconsistency of the initial and boundary condition on ${\varGamma _\textrm{D}}$ makes the computation quite difficult for $t\approx 0$.

Figure 3b verifies the conservativity of the adaptive method. We plot the quantities

106 $$\begin{aligned} F(t)&= \int _{0}^t \int _{\varGamma }{\textbf{K}}(u) \nabla u\cdot n{\,{\mathrm d}S}{\,{\mathrm d}t},\nonumber \\ \varDelta V(t)&= V(t) - V(0),\ \ V(t) = \int _{\varOmega }{\vartheta }(u(\cdot ,t)) {\,{\mathrm d}x},\ t\in [0,T], \end{aligned}$$

where *F*(*t*) is the total flux of the water through the boundary ${\varGamma }$ till time *t* and $\varDelta V(t)$ is the changes of the water content in the domain between times 0 and *t*. From equation (3) and the Stokes theorem, we have the conservation law $F(t) = \varDelta V(t)$ for all $t\in [0,T]$. Therefore, we also show the relative difference between these quantities $|F(t) - \varDelta V(t)|/ \varDelta V(t)$ for $t>0$ in Fig. 3b the vertical label on the right. We observe that, except for the time close to zero, where the inconsistency between initial and boundary conditions is problematic, the relative difference is at the level of several percent.

**Fig. 4:**
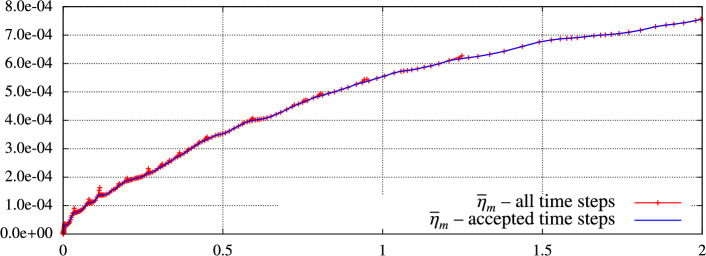
Single ring infiltration, performance of Algorithm 1, accumulated error estimator ${\overline{\eta }}_m$ with respect to $t_m$, $m=1,\dots ,r$

Furthermore, Fig. 4 shows the accumulated estimators ${\overline{\eta }}_m =\sum _{i=1}^m \eta _i$ for time levels $t_m$, $m=1,\dots ,m$. The red nodes correspond to all computed time steps, including the rejected steps whereas the blue line connects only the accepted time steps. The rejected time steps are followed by the remeshing which is carried out namely for small *t*. We observe that the elimination of the rejected time steps causes that the errors arising from the remeshing do not essentially affect the total error estimate $\eta$.

**Fig. 5:**
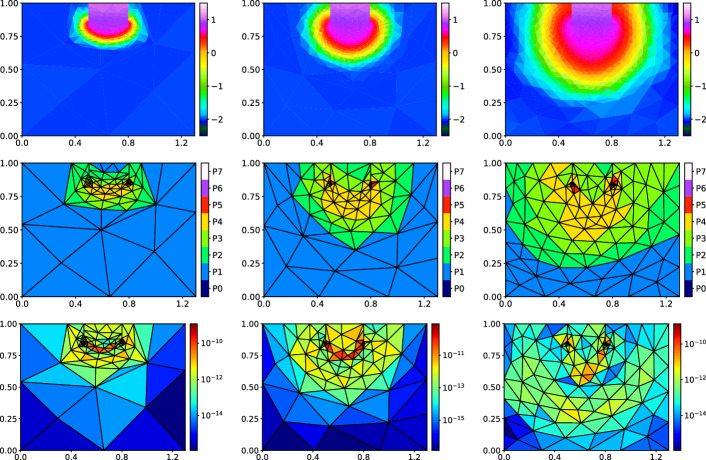
Single ring infiltration, hydraulic head (top ), the corresponding *hp*-meshes obtained by Algorithm 1 (center) and the error estimators $\eta _{K,m}$, $K\in {{\mathcal {T}}_h^m}$ (bottom) at $t=0.4$, $t=0.8$ and $t=2$ $\,\textrm{hours}$ (from left to right)

Moreover, Fig. 5 shows the *hp*-meshes, the hydraulic head and the error estimator $\eta _{K,m}$, $K\in {{\mathcal {T}}_h^m}$ at selected time levels obtained from Algorithm 1 with $\delta =5.0 \times 10^{-3}$. We observe the mesh adaptation namely at the (not sharp) interface between the saturated and non-saturated medium and also in the vicinity of the domain singularities. The error estimators $\eta _{K,m}$, $K\in {{\mathcal {T}}_h^m}$ indicate an equi-distribution of the error.

## Conclusion

We derived reliable and efficient a posteriori error estimates in the residual-based norm for the Richards equation discretized by the space-time discontinuous Galerkin method. The numerical verification indicates the effectivity indexes between 1 and 2.5 for the tested examples. Moreover, we introduced the *hp*-mesh adaptive method handling varying non-nested and non-matching meshes and demonstrated its efficiency for simple test benchmark and its applicability for the numerical solution of the single ring infiltration experiment.

It will be possible to generalize the presented approach to genuinely space-time *hp*-adaptive method, where the (local) polynomial order *q* in time is varied as well. However, the question is of potential benefit. Based on our experience, the setting $q=1$ gives sufficiently accurate approximation for the majority of tested problems.

On the other hand, the choice $q=0$ would be sufficient only in subdomains of ${\varOmega }$ where the solution is almost constant in time. Therefore, we suppose that the benefit of local varying of polynomial order in time will be low.

Although the presented numerical examples are two-dimensional, it would be possible to apply the presented error estimates and mesh adaptation to three-dimensional problems as well. We refer, e.g., to [1] and the references therein, where the anisotropic mesh adaptation techniques are developed for time-dependent 3D problems.

## Data Availability

No datasets were generated or analysed during the current study.
